# Chemical synthesis of tripeptide thioesters for the biotechnological incorporation into the myxobacterial secondary metabolite argyrin via mutasynthesis

**DOI:** 10.3762/bjoc.15.286

**Published:** 2019-12-05

**Authors:** David C B Siebert, Roman Sommer, Domen Pogorevc, Michael Hoffmann, Silke C Wenzel, Rolf Müller, Alexander Titz

**Affiliations:** 1 Chemical Biology of Carbohydrates, Helmholtz Institute for Pharmaceutical Research Saarland (HIPS), Helmholtz Centre for Infection Research (HZI), D-66123 Saarbrücken, Germany; 2 Deutsches Zentrum für Infektionsforschung (DZIF), Standort Hannover-Braunschweig, Germany; 3 Microbial Natural Substances, Helmholtz Institute for Pharmaceutical Research Saarland (HIPS), Helmholtz Centre for Infection Research (HZI), D-66123 Saarbrücken, Germany; 4 Department of Pharmacy, Saarland University, D-66123 Saarbrücken, Germany

**Keywords:** antibiotic, argyrin, mutasynthesis, NRPS, peptide synthesis

## Abstract

The argyrins are secondary metabolites from myxobacteria with antibiotic activity against *Pseudomonas aeruginosa*. Studying their structure–activity relationship is hampered by the complexity of the chemical total synthesis. Mutasynthesis is a promising approach where simpler and fully synthetic intermediates of the natural product’s biosynthesis can be biotechnologically incorporated. Here, we report the synthesis of a series of tripeptide thioesters as mutasynthons containing the native sequence with a dehydroalanine (Dha) Michael acceptor attached to a sarcosine (Sar) and derivatives. Chemical synthesis of the native sequence ᴅ-Ala-Dha-Sar thioester required revision of the sequential peptide synthesis into a convergent strategy where the thioester with sarcosine was formed before coupling to the Dha-containing dipeptide.

## Introduction

Resistance to antibiotics is currently a major threat to public health. Especially Gram-negative bacterial pathogens are of concern, due to their widespread development of resistance mechanisms. To address this general antimicrobial resistance problem, new variants of known antibiotics are being developed and were approved in the last few years, also comprising drugs active against *Pseudomonas aeruginosa*, one of the currently most problematic bacterial pathogens [1]. Especially among the quinolones, cephalosporins and carbapenems new compounds have been identified. In addition, the development of new β-lactamase inhibitors is ongoing and may restore the activity of known β-lactams against β-lactamase-producing strains [2]. Unfortunately, most of these antibiotics rely on known modes of action and do not target novel binding sites. To circumvent established resistances in clinically used bacterial targets, innovative antiinfective strategies comprising new antibiotic classes and virulence-attenuating compounds have been developed [3–4].

**Figure 1:**
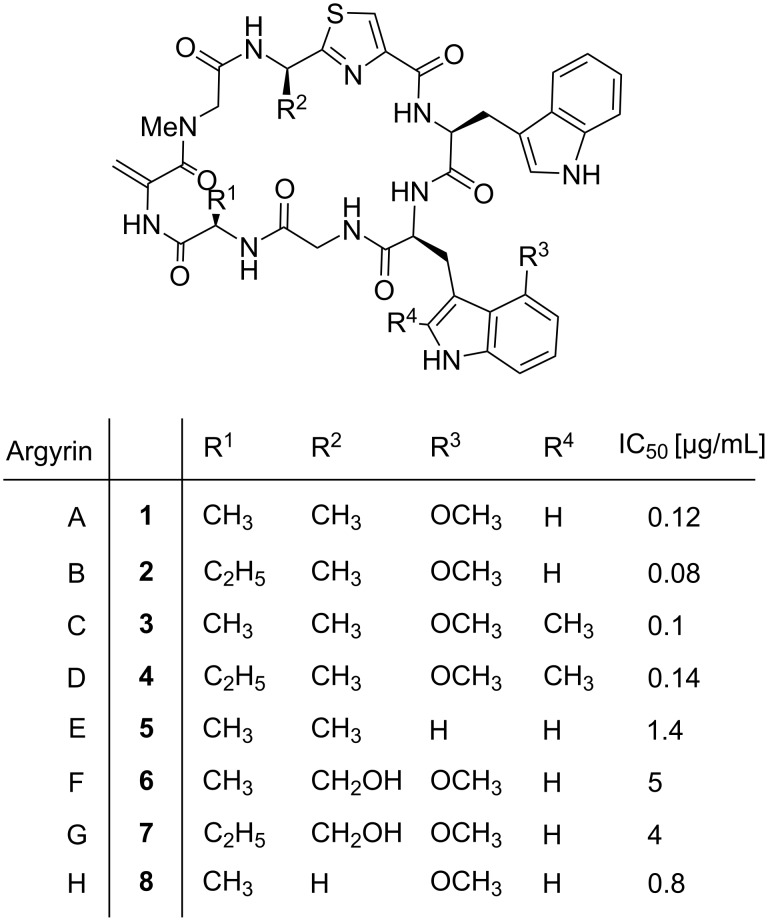
Chemical structures of naturally occurring argyrins with potent antipseudomonal activity.

The argyrins **1–8** are a family of 8 naturally occurring cyclic peptides isolated by Sasse, Höfle and co-workers from the myxobacterium *Archangium gephyra* (Figure 1) [5–6]. These cyclic peptides have interesting biological activities such as cytotoxic activity presumably via proteasome inhibition and immunomodulatory effects, and they also show good antibiotic effects against *P. aeruginosa* [7–9]. The structure–activity relationship for the natural argyrins A–H revealed argyrin B as most potent derivative (**2**, IC_50_ 0.08 µg/mL). In 1996, two cyclic peptides with a similar sequence but a proposed regioisomer of the methoxytryptophan were reported as antibiotics A21459A and B [7,10]. Later, it was shown that A21459A and B are identical to argyrin A and B, respectively, and their structure was revised with respect to the position of the methoxy substituent [6]. Argyrin A demonstrated a high efficacy against a panel of *P. aeruginosa* multidrug resistant (MDR) strains and the argyrins were shown to bind to elongation factor G (EF-G, encoded by the gene fusA1) as their target [11–12]. The co-crystal structure of argyrin B (**2**) and *P. aeruginosa* EF-G1 provides structural information of the complex at atomic resolution as basis for further structure-based optimization [11]. Jones and colleagues analyzed possible resistance mechanisms to argyrin and established argyrin B as an efflux substrate. Further, they showed that in susceptible *Stenotrophomonas maltophilia*, resistance appeared by mutational inactivation of the gene fusA1 and overexpression of the alternative elongation factor FusA2 [13]. Due to the dual activity spectrum, cytotoxicity and antibiotic activity, the selectivity profile of this class of compounds should be considered for optimization of future antibacterials.

The first total synthesis was reported by Ley and co-workers [14] for argyrin B in 18 linear steps, followed by an alternative strategy towards argyrin F (**6**) by the Kalesse group [15]. In 2011, Wu et al*.* reduced the length to 14 linear steps for the total synthesis of argyrins A and E [16]. Finally, Chen et al*.* described a synthesis yielding several derivatives of argyrin A with modification of the 4’-methoxytryptophan residue [17]. Changing the 4’-methoxy group to halogens or other substituents in different positions led to a loss of antibacterial activity, whereas the 5’-methoxytryptophan regioisomer largely retained activity against *P. aeruginosa* PAO1.

Inspection of the crystal structure of argyrin in complex with the bacterial elongation factor G reveals that the antibiotic is deeply buried inside the protein’s binding pocket and only a few sites of the molecule can possibly be modified without introducing steric clash with the binding site. Besides the methoxytryptophan which has been addressed by chemical synthesis, one appealing possibility resides in the dehydroalanine-sarcosine motif and modification seems possible as deduced from the crystal structure [11] of argyrin with elongation factor G1.

Despite the successful implementation of total syntheses of argyrin derivatives, a more rapid access towards diversity in the argyrins and the resulting structure–activity relationship knowledge is desirable. Besides the chemical total synthesis, argyrins can be obtained from the producer organism in sufficient amounts and purity but lack the diversity desired for an extended structure–activity relationship to develop lead candidates. It has been observed in many cases that exogenous substrates can be incorporated by bacteria into biosynthesis cascades of natural products. The use of substrates which lead to nonnatural derivatives of the natural product coined the field of mutasynthesis, e.g., siderophore analogue biosynthesis by *P. aeruginosa* [18]. Biotechnological engineering of producer strains aims to shutdown the natural substrate production and thereby increase the usually poor yields of the mutasynthesis products [19–20]. For bacterial natural products that originate from a polyketide synthase (PKS) or a nonribosomal peptide synthetase (NRPS), mutasynthons often carry thioesters to mimic the natural phosphopantetheinyl conjugate [20].

**Figure 2:**
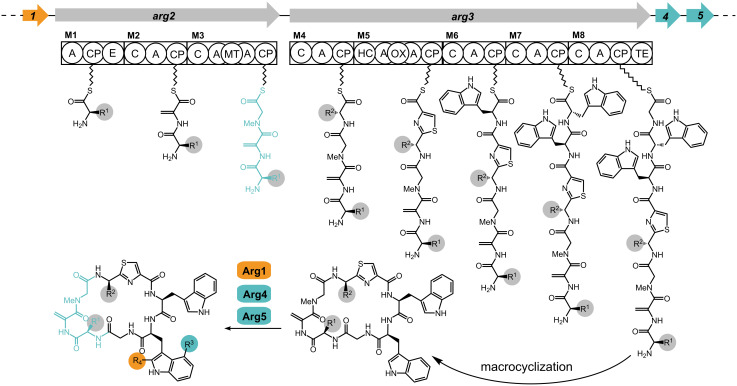
The biosynthetic pathway for argyrin production in *Cystobacter sp.* SBCb004 (Arg1, radical SAM-dependent methyltransferase; Arg2/Arg3, nonribosomal peptide synthetases; Arg4, O‑methyltransferase; Arg5, tryptophan 2,3-dioxygenase). The initial tripeptide of the biosynthesis of the argyrins, i.e., the target for the mutasynthons of this work, is color coded as product of the Arg2 synthetase and in the resulting final argyrin molecule.

For the argyrins, the Müller group identified the corresponding biosynthetic gene cluster from *Cystobacter sp.* SBCb004 [21], studied the biosynthesis (Figure 2) and established a heterologous expression system for the entire pathway in a derivative of *Myxococcus xanthus* DK1622 [22]. On this basis, a mutant strain for the mutasynthesis approach (*M. xanthus* DK1622 *∆mchA-tet*::pArg345-V1) lacking the genes *arg1* and *arg2* was constructed. This was designed to enable a biosynthetic production of argyrin derivatives upon incorporation of synthetically provided tripeptide thioester intermediates, the so called mutasynthons. Mutasynthons are synthesized as SNAc thioesters which mimic the phosphopantetheine (PPant) moiety normally present on the PCP domain of the NRPS. It has been shown by several mutasynthesis studies [20], that SNAc thioesters can serve as a mimic of PPant and thus get accepted by the subsequent C domain which forms the peptide bond with the downstream building block. Because it lacks *arg2*, the carrier-associated tripeptide produced by the first NRPS subunit is not available for biosynthesis. This fact provides the opportunity to chemically substitute the initial tripeptide thioester accepted by module 4 on the Arg3 subunit and replace the natural construct in biosynthesis. By feeding the block mutant with synthetic analogs, derivatives should be accessible without the need for full total synthesis of this complex natural product (Supporting Information File 1, Figure S1).

## Results and Discussion

**Figure 3:**
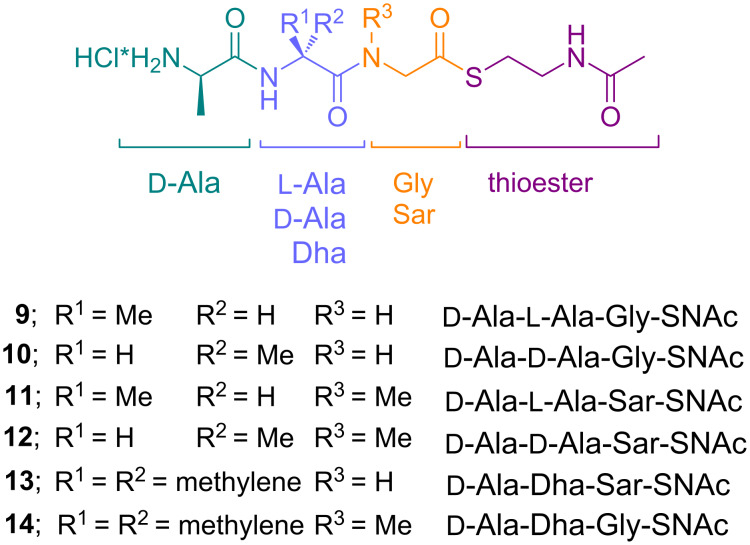
Designed mutasynthons **9**–**14** for argyrin biosynthesis. Peptides are based on three amino acids and additionally bear the thioester moiety mimicking the native phosphopantetheinyl arm of the peptide carrier protein (CP).

**Scheme 1:**
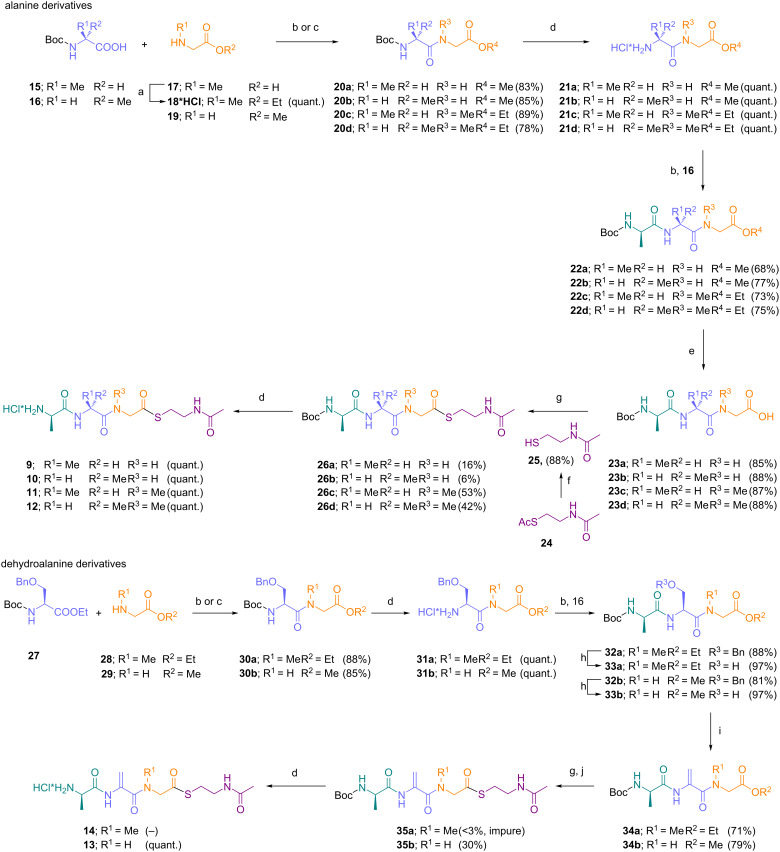
Synthesis of tripeptide thioesters. Reagents and conditions: (a) SOCl_2_, EtOH, 78 °C; (b) IBCF, NMM, THF, −20 °C (for **20a, 20b**, **30a**); (c) PyBOP, DIPEA, CH_2_Cl_2_, 0 °C (for **20c**, **20d**, **30b**); (d) 4 M HCl/dioxane, rt; (e) NaOH, dioxane, 0 °C; (f) KOH, H_2_O, 0 °C to rt; (g) (for **26a**) HSCH_2_CH_2_NHAc (HSNAc), IBCF, NMM, THF, −20 °C; (for **26b–d, 35a,b**) HSNAc, TFFH, DIPEA, CH_2_Cl_2_, 0 °C; (h) Pd/C, H_2_, MeOH, rt; (i) EDC, CuCl, CH_2_Cl_2_, rt; (j) 0.5 M LiOH, THF/MeOH/H_2_O (4:1:2), 0 °C to rt.

In order to establish the mutasynthesis of argyrins, we designed the native sequence mutasynthon **14** and various synthetic analogs of this initiating tripeptide for argyrin mutasynthesis with relatively small structural changes to probe the biosynthetic machinery: the native sequence ᴅ-alanine-dehydroalanine-sarcosine was varied in a small library by replacing dehydroalanine (Dha) with ᴅ- or ʟ-alanine and sarcosine with glycine (Figure 3). The synthesis of these biosynthesis analogs **9**–**14** was performed following solution phase Boc-protected peptide coupling and functional group interconversion (Scheme 1).

The central alanine carrying nonnatural derivatives were sequentially synthesized from C- to N-terminus, using ester protected glycine or sarcosine and coupling to Boc-protected ᴅ- or ʟ-alanine, acid-mediated deprotection of the Boc group and final coupling to Boc-ᴅ-alanine. Fully protected tripeptides were transformed by base hydrolysis into the free carboxylic acids, followed by activation of the unprotected C-terminus as a SNAc thioester. Subsequent cleavage of the N-terminal Boc protecting group gave the unnatural analogs containing ᴅ- or ʟ-alanine at the dehydroalanine site and sarcosine or glycine at the C-terminus (**9–12**) in generally acceptable yields.

The synthesis of the dehydroalanine containing analogs **13** and **14** was performed following the same strategy with incorporation of a benzyl protected serine at the Dha site. Following hydrogenolytic deprotection, in situ produced serine EDC adduct was subjected to copper(I) chloride-mediated elimination to give the dehydroalanine derivatives **34a** and **34b** in good yields. Deprotection of the ester protecting group also proceeded in good yields. The subsequent activation as thioesters (→**35a** and **35b**, respectively) proved difficult due to the reactivity of the dehydroalanine moiety. Therefore, only moderate yields were obtained for the glycine analog **13**, whereas no product of the analog **14** carrying the native sequence containing the sarcosine moiety could be obtained.

**Scheme 2:**
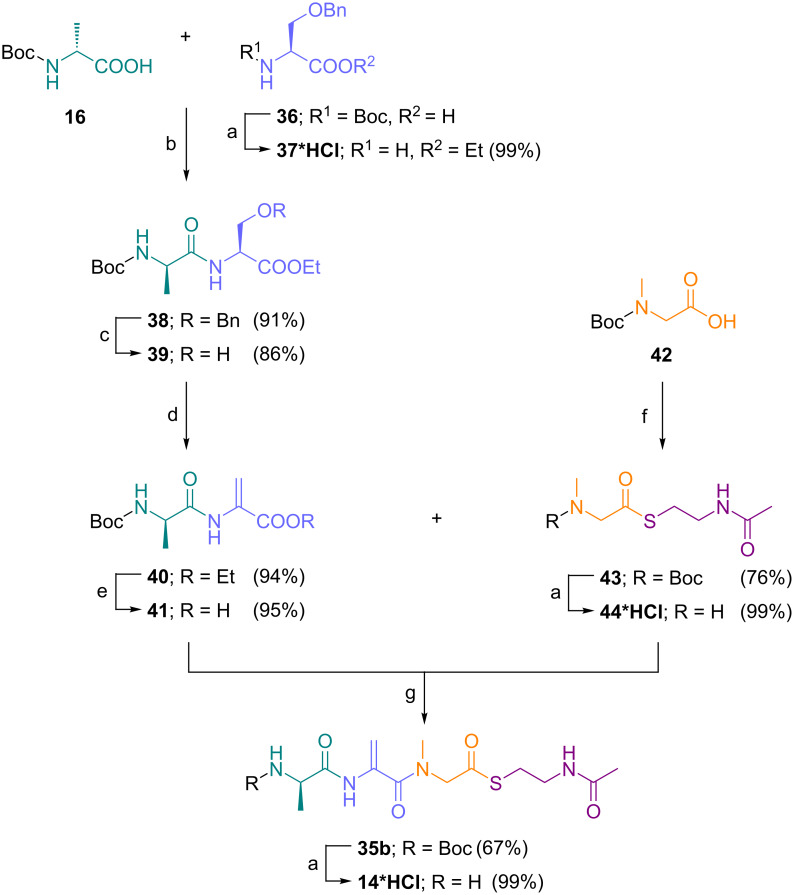
Improved synthesis of the tripeptide thioester **14**. Reagents and conditions: (a) SOCl_2_, EtOH, 78 °C; (b) IBCF, NMM, THF, −20 °C; (c) Pd/C, H_2_, MeOH, rt; (d) EDC, CuCl, CH_2_Cl_2_, rt; (e) 0.5 M LiOH, THF/H_2_O (4:1:2), 0 °C to rt; (f) HSCH_2_CH_2_NHAc, TFFH, DIPEA, CH_2_Cl_2_, 0 °C; (g) BOP-Cl, DIPEA, CH_2_Cl_2_, 0 °C.

**Figure 4:**
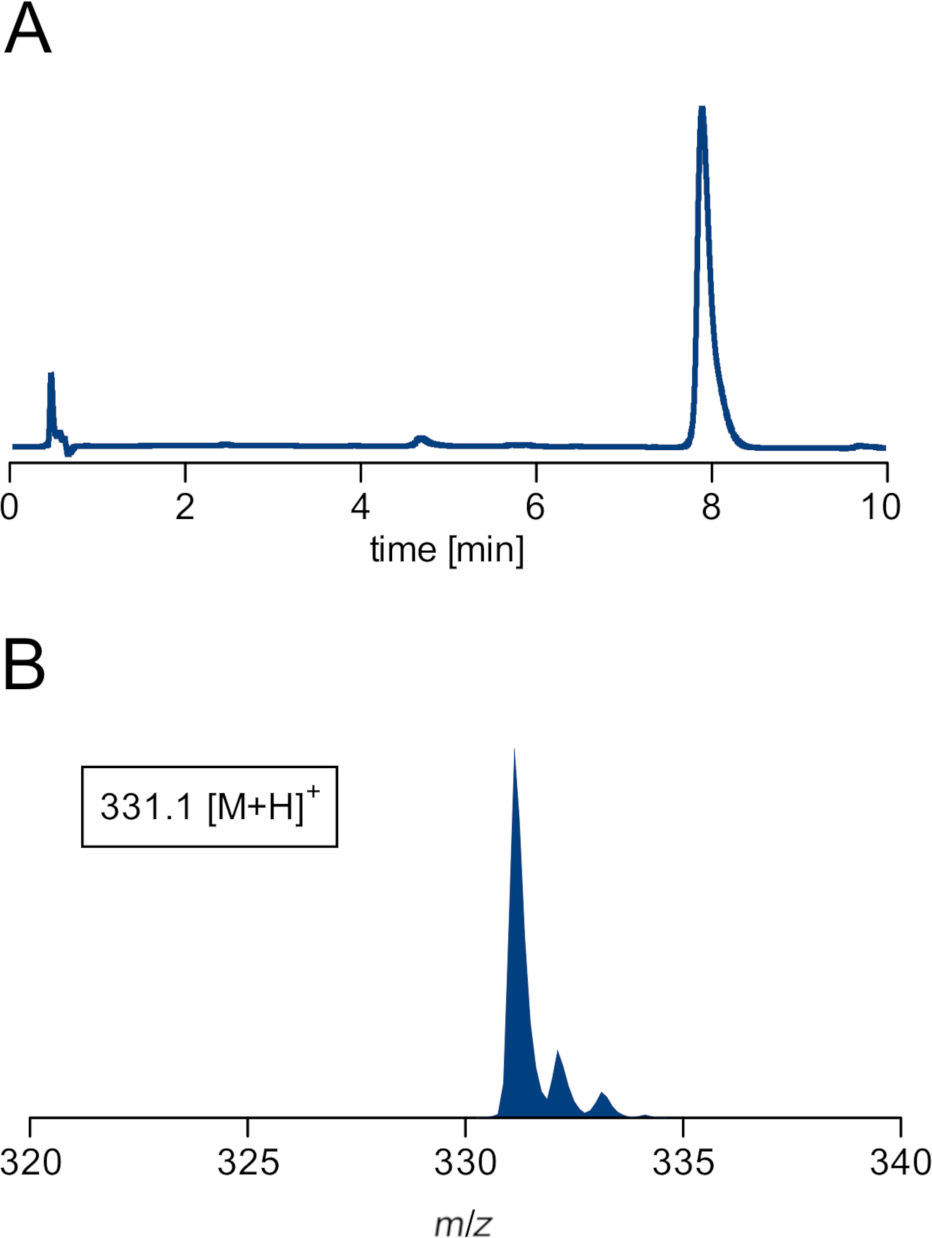
Analysis of mutasynthon **14** obtained via the convergent synthetic route by HPLC on a HILIC stationary phase and UV detection (A) and mass spectrometry (B).

To overcome the high reactivity of the Dha moiety towards nucleophiles, we engaged in an alternative synthetic strategy towards the tripeptide thioester carrying the native sequence ᴅ-Ala-Dha-Sar by designing a convergent synthesis to mutasynthon **14** (Scheme 2). First, Boc-ᴅ-Ala (**16**) was coupled to O-benzylserine ethyl ester **37** to give dipeptide **38**, hydrogenolytic debenzylation gave the substrate **39** for the copper(I)-mediated elimination of the carbodiimide formed in situ using EDC, and then yielded the dipeptide Boc-ᴅ-Ala-Dha ester **40** in good yields. Lithium hydroxide deprotection of the ester in compound **40** provided the free acrylate **41**. In parallel, Boc-protected sarcosine **42** was transformed into the SNAc thioester **43** and the Boc group was removed to give **44**. Then, both building blocks, **44** and **41**, were coupled to give the protected tripeptide thioester **35b** in good yields. The mutasynthon **14** was obtained after Boc-deprotection from this convergent synthetic approach in good yields and sufficient quantities for testing its biotechnological incorporation into argyrin (Figure 4).

In a first mutasynthesis experiment, the 5 nonnatural derivatives **9–13** were tested for biotechnological incorporation into argyin by using the *arg1*/*arg2*-deficient producer strain. Although thioesters have been successfully used for incorporation in different mutasynthesis projects [20], the feeding experiment using **9–13** for argyrin biosynthesis unfortunately resulted in no detectable incorporation of these nonnatural mutasynthons. The reasons for failure remain obscure and it could not be excluded that the argyrin biosynthetic machinery is highly specific and requires the natural substrate analog, i.e., the sequence ᴅ-Ala-Dha-Sar. Furthermore, it is not clear whether uptake of these tripeptide-thioesters into the bacterial cell was insufficient or compound stability under microbiological growth conditions is insufficient.

Mutasynthons representing mimics of the native biosynthetic intermediate can be used as proof of concept in mutasynthesis studies, to show that production of the native compound can be restored [23]. Following this example, incorporation using the ᴅ-Ala-Dha-Sar thioester **14** was performed. Surprisingly, also in this experiment, incorporation could not be detected. In general, advanced mutasynthesis studies relying on complex biosynthetic intermediates are quite challenging, e.g., as shown in previous studies on polyketide chain engineering in α-pyrone antibiotic biosynthesis [23]. We assumed that the insufficient/missing processing of the SNAc precursors by the NRPS subunit Arg3 is the main culprit for unsuccessful restoration of the argyrin production. As previously mentioned, one reason for this could be high specificity of the biosynthetic machinery which might require a full length phosphopantetheine moiety or even a PCP bound substrate, for condensation to take place.

To verify the functionality of the truncated argyrin biosynthesis operon (*arg3*-*arg4*-*arg5*) gene *arg2* was integrated into the host chromosome at a different locus. The obtained mutant strain *M. xanthus* DK1622 ∆*mchA-tet*::pArg345-V1-nptII-arg2 was shown to produce argyrins indicating that the engineered operon is functionally expressed via the integrated pArg345-V1 construct.

One further possibility for the lack of successful incorporation into argyrins is an intrinsically limited chemical stability of compound **14** which possesses a thioester function and a Michael acceptor motif. Stability of the ᴅ-Ala-Dha-Sar thioester **14**, was evaluated by measuring its degradation kinetics under the standard conditions in the cultivation medium. The HPLC–MS analysis revealed a rather rapid degradation of the analysed mutasynthon, as after only 2 h of incubation time the majority was degraded and after 4 h of incubation almost complete degradation of the compound was observed (Supporting Information File 1, Figure S2).

To exclude the possibility of inefficient mutasynthon permeability through the bacterial membrane, we decided to repeat the mutasynthesis experiment in vitro using the cell lysate of the respective *arg2* deletion mutant. The in vitro reconstitution of nonribosomal peptide biosynthesis has previously been successfully performed using cell lysate, e.g., in case of gramicidin [24], or with purified proteins, e.g., in case of myxochelin [25] and tilivalline [26]. To evaluate if in vitro formation of argyrin is indeed possible, the reaction buffer was incubated with the cell lysate mixture of *M. xanthus* DK1622 mutants expressing the Arg2-Arg5 argyrin biosynthetic proteins. To ensure that no argyrin is present in the lysate prior to the incubation, a lysate mixture of two different mutants harboring *arg2* and *arg3-5* genes was used, respectively. Both of the mutants are incapable of argyrin production on their own, however, together they express all the necessary proteins. Despite significant efforts and evaluation of various incubation conditions, in vitro reconstitution of argyrins could never be achieved. The exact reason for this is unknown, however, it is very likely that one or more of the biosynthetic proteins are not active under the applied conditions. Experimental details are provided in Supporting Information File 1.

## Conclusion

In summary, we have designed and successfully synthesized a library of tripeptide thioesters for the use in the mutasynthesis of argyrin derivatives. In the initial strategy, the sequential synthesis of the peptides followed by activation as a SNAc ester successfully yielded five out of six desired library members. Following this strategy, the mutasynthon carrying the native sequence ᴅ-Ala-Dha-Sar could not be transformed into the desired thioester probably due to the higher reactivity of the Michael acceptor system. Revision of the synthetic approach into a convergent synthesis with initial formation of the thioester at the amino acid level and subsequent coupling to a dipeptide finally yielded the desired mutasynthon carrying the native sequence in high amounts and purity. Reasons for failure to incorporate these mutasynthons even at analytical scale remain obscure and may lie in a tight substrate specificity, a high rate of degradation of the highly reactive native sequence ᴅ-Ala-Dha-Sar thioester, and/or insufficient cell permeability of the mutasynthons.

## Experimental

Protocols and methods can be found in Supporting Information File 1.

## Abbreviations

Boc: *tert-*Butyloxycarbonyl, BOP-Cl: bis(2-oxo-3-oxazolidinyl)phosphinic chloride, CDI: carbonyldiimidazole, DIPEA: diisopropylethylamine, EDC: *N*-(3-Dimethylaminopropyl)-*N*′-ethylcarbodiimide hydrochloride, IBCF: isobutyl chloroformate, NMM: *N*-methylmorpholine, PyBOP: (benzotriazol-1-yloxy)tripyrrolidinophosphonium hexafluorophosphate, SNAc: SCH_2_CH_2_NAc, TFFH: fluoro-*N*,*N*,*N*′,*N*′-tetramethylformamidinium hexafluorophosphate.

## Supporting Information

File 1 Experimental part.
