# Supplementary material for: Chemical synthesis of tripeptide thioesters for the biotechnological incorporation into the myxobacterial secondary metabolite argyrin via mutasynthesis
Source: Beilstein J Org Chem. 2019 Dec 5;15:2922–9. doi: 10.3762/bjoc.15.286 (PMC6902895; doi:10.3762/bjoc.15.286)
Supplement: File 1 — Experimental part. [file Beilstein_J_Org_Chem-15-2922-s001.pdf]

BEILSTEIN JOURNAL OF ORGANIC CHEMISTRY

Supporting Information

for

Chemical synthesis of tripeptide thioesters for the biotechnological incorporation into the myxobacterial secondary metabolite argyrin via mutasynthesis

David C. B. Siebert, Roman Sommer, Domen Pogorevc, Michael Hoffmann, Silke C. Wenzel, Rolf Müller and Alexander Titz

Beilstein J. Org. Chem. **2019,** *15,* 2922–2929. doi:10.3762/bjoc.15.286

Experimental part

License and Terms: This is a supporting information file under the terms of the Creative Commons Attribution License (http://creativecommons.org/licenses/by/4.0). Please note that the reuse, redistribution and reproduction in particular requires that the authors and source are credited.
The license is subject to the *Beilstein Journal of Organic Chemistry* terms and conditions: (https://www.beilstein-journals.org/bjoc)

Experimental details

Chemical synthesis

Silica gel 60 coated aluminum sheets containing fluorescence indicator (Merck KGaA, Darmstadt, Germany) were used for thin layer chromatography (TLC). UV light (254 nm) and aqueous $KMnO_4$ solution or a molybdate solution (0.02 M ammonium cerium sulfate dihydrate and 0.02 M ammonium molybdate tetrahydrate in aqueous 10% H_2SO_4) were used for development. Medium pressure liquid chromatography (MPLC) was performed on a Teledyne Isco Combiflash Rf200 system using pre-packed silica gel 60 columns from Teledyne Isco, SiliCycle or Macherey-Nagel. Nuclear magnetic resonance (NMR) spectroscopy was performed on a Bruker Avance III 500 UltraShield spectrometer at 500 MHz (^{1}H) or 126 MHz (^{13}C) or a Bruker Fourier300 spectrometer at 300 MHz (^{1}H) or 75 MHz (^{13}C). Chemical shifts are given in ppm and were calibrated on residual solvent peaks as internal standard. Multiplicities were specified as s (singlet), d (doublet), t (triplet) or m (multiplet). The signals were assigned with the help of ^{1}H, ^{1}H - COSY, DEPT-135-edited ^{1}H, ^{13}C-HSQC and ^{1}H, ^{13}C-HMBC experiments. Mass spectra were obtained on Thermo Dionex 3000 HPLC frontend coupled to a Bruker amaZon SL and the data were analyzed using DataAnalysis from Bruker (Bremen, Germany) or on a Thermo SpectraSystem HPLC frontend coupled to a Finnigan Surveyor MSQ Plus.

High resolution mass spectra (HRMS) for compound **14** were obtained on a Bruker maxis 2G hr-qToF spectrometer after separation on a HILIC column (see below), and the data were analyzed using DataAnalysis (Bruker Daltonics, Bremen, Germany). The HRMS measurements for compounds **9-13** were conducted on a solariX XR (7T) FT-ICR mass spectrometer (Bruker Daltonics, Germany) using the Apollo ESI source. Samples were injected with the pre-installed syringe pump at a concentration of approx. 0.1 mg/mL in a mixture of water/ACN (1:1). In the source region, the temperature was set to 220 °C, the capillary voltage was 4500 V, the dry-gas flow was 4.0 L/min and the nebulizer to 1.0 bar. Mass spectra were acquired in positive ionization mode ranging from 50–1000 *m*/*z* with a FID data size of 4M and 20 ms accumulation in the quadrupole. For each mass spectrum, 8 scans were combined.

Commercial chemicals and solvents were used without further purification. Deuterated solvents, *N*-Boc-sarcosine, sarcosine, *N*-Boc-Ser(OBn)-OH and *N*-acetylcysteamine were purchased from Sigma-Aldrich (Steinheim, Germany). *N*-Boc-D-alanine was purchased from Iris Biotech GmbH (Marktredwitz, Germany); EDC and fluoro-*N,N,N',N'*-tetramethylformamidinium hexafluorophosphate (TFFH) from Carbosynth (Berkshire, United Kingdom); DIPEA and PyBOP from Carl Roth GmbH (Karlsruhe, Germany). Isobutyl chloroformate (IBCF), bis(2-oxo-3-oxazolidinyl)phosphinic chloride (BOP-Cl), NMM, glycine methyl ester hydrochloride and 4 M HCl in 1,4-dioxane were purchased from Acros Organics (Geel, Belgium). *N*-Boc-L-alanine was purchased from Carbolution GmbH (St. Ingbert, Germany).

***N*-Acetylcysteamine (25).** *N,S*-Diacetylcysteamine (**24**) (150 mg, 0.93 mmol) was dissolved in H_2O (1.2 mL), cooled to 0 °C and the solution degassed with N_2. After addition of KOH (165 mg, 3.07 mmol) the reaction mixture was stirred for 40 min at rt. The solution was neutralized with 2 N HCl, saturated with solid NaCl and extracted with CH_2Cl_2 (3 × 2 mL). The combined organic layers were dried over Na_2SO_4, filtered and the solvent was removed under reduced pressure to give *N*-acetylcysteamine (**25**, 97 mg, 0.81 mmol, 88%) as colorless oil. ^{1}H NMR (500 MHz, $CHCl_3$-*d1*) δ 6.55 (s, 1H, NH), 3.39 (q, J = 6.3 Hz, 2H, NCH2), 2.64 (dt, J = 8.4, 6.5 Hz, 2H, SCH2), 1.99 (s, 3H, CH3), 1.39 (t, J = 8.4 Hz, 1H, SH). ^{13}C NMR (126 MHz, $CHCl_3$-*d1*) δ 170.71 (CONH), 42.67 (NCH2), 24.53 (SCH2), 23.15 (CH3). Proton NMR data correspond to the literature values [1].

Sarcosine ethyl ester hydrochloride (Sar-OEt * HCl) (18). To a solution of sarcosine (**17**) (1.0 g, 11.2 mmol) in EtOH (26 mL) $SOCl_2$ (2.44 mL, 33.6 mmol) was added drop-wise at 0 °C. The reaction mixture was heated to reflux, kept overnight and the solvent was then removed under reduced pressure to give sarcosine ethyl ester (**18,** 1.72 g, 11.2 mmol, quant.) as white solid. ^{1}H NMR (500 MHz, MeOH-d_4) δ 4.31 (q, J = 7.1 Hz, 2H, OCH2CH3), 3.96 (s, 2H, Sar-CH2), 2.76 (s, 3H, N-CH3), 1.32 (t, J = 7.2 Hz, 3H, OCH2CH3). ^{13}C NMR (126 MHz, MeOH-d_4) δ 167.63 (CO), 63.53 (OCH2CH3), 49.79 (Sar-CH2), 33.63 (N-CH3), 14.34 (OCH2CH3).

General Operation Procedures (GOP):

GOP A: Coupling of Sar-OEt * HCl:
PyBOP (1.1 equiv) was dissolved in CH_2Cl_2 (1.2 mL/mmol) and cooled to 0 °C. After addition of the Boc-protected amino acid (1.1 equiv) and DIPEA (2.4 equiv) the solution was stirred at 0 °C for 10 min. Sarcosine ethyl ester hydrochloride (1 equiv) was added portion-wise and the reaction mixture was allowed to warm to room temperature. After 2 h the reaction mixture was diluted with EtOAc and subsequently washed with aqueous satd. $NaHSO_3$, satd. $NaHCO_3$ and brine three times each. The organic layer was dried over Na_2SO_4, filtered and the solvent was removed under reduced pressure. The residue was purified by flash column chromatography (PE with linear gradient of EtOAc 5% to 100%) to give the *N*-methylated dipeptide.

GOP B: Peptide coupling via mixed anhydride:
To a solution of the carboxylic acid (1 equiv) in THF (10 mL/mmol) and NMM (1.2 equiv) was added IBCF (1.1 equiv) at −20 °C. The solution was stirred for 10 min and in case of the hydrochloride salt of the amine NMM (1.2 equiv) was added again. Free amines (1.1 equiv) were added dropwise (dissolved in THF 15 mL/mmol) and the reaction mixture was stirred overnight and allowed to warm to rt. The reaction mixture was diluted with EtOAc and subsequently washed with aqueous satd. $NaHSO_3$, satd. $NaHCO_3$ and brine three times each. The organic layer was dried over Na_2SO_4, filtered and the solvent was removed under reduced pressure. The residue was purified by flash column chromatography (PE with linear gradient of EtOAc 5% to 100%) to give the amide product.

GOP C: Removal of Boc protecting group:

10 equiv of HCl in dioxane (4 M solution) were added to 1 equiv of the solid Boc protected peptide at room temperature. After 0.5–1 h the reaction was completed and the solvent was removed under reduced pressure.

GOP D: Removal of benzyl protecting group

The benzyl protected peptide (1 equiv) was dissolved in MeOH (10 mL/mmol) and Pd/C (10 wt %) was added. The suspension was stirred overnight under a H_2-atmosphere. The reaction mixture was filtered through celite and the solvent was removed under reduced pressure.

GOP E: Alkaline ester hydrolysis:

The ester (1 equiv) was dissolved in dioxane (5 mL/mmol), 1.1 equiv of an aqueous 1 M NaOH solution was added at 0 °C under stirring and after 1 h the solvent was removed under reduced pressure. The residue was redissolved with H_2O (40 mL/mmol) and acidified with 2 M HCl to pH = 2. The aqueous phase was extracted with EtOAc (3 × 50 mL/mmol), the combined organic layers were dried over Na_2SO_4, filtered and the solvent was removed under reduced pressure to give the free acid.

GOP F: Synthesis of tripeptide SNAc-ester:

To a solution of the carboxylic acid (1 equiv) in CH_2Cl_2 (15 mL/mmol) was added DIPEA (2 equiv) and the solution was cooled to 0 °C. After addition of TFFH (1.1 equiv) the solution was stirred for 15 min at 0 °C. *N*-acetylcysteamine (1.1 equiv) was added portion-wise and the reaction mixture was stirred at 0 °C until the reaction was completed (TLC). The reaction mixture was diluted with EtOAc (150 mL/mmol), washed with satd. $NaHCO_3$ (3 × 75 mL/mmol) and with brine (1 × 75 mL/mmol). The organic layer was dried over Na_2SO_4, filtered and the solvent was removed under reduced pressure. Purification by flash column chromatography (linear gradient of CH_2Cl_2/EtOH 0–7%) gave the SNAc esters.

***N*-Boc-L-Ala-Gly-OMe (20a)**. The compound was synthesized according to GOP B using Boc-L-Ala-OH (189 mg, 1.0 mmol), Gly-OMe · HCl (138 mg, 1.1 mmol), IBCF (142 µL, 1.1 mmol), NMM (132 µL, 1.2 mmol). Purification gave Boc-L-Ala-Gly-OMe (**20a**) (216 mg, 0.83 mmol, 83%) as colorless oil. ^{1}H NMR (500 MHz, $CHCl_3$-*d1*) δ 6.81 (br-s, 1H, NH), 5.10 (br-d, J = 7.6 Hz, 1H, NH), 4.23 (br-s, 1H, CH), 4.03 (t, J = 4.8 Hz, 2H, Gly-CH2), 3.74 (s, 3H, OCH3), 1.43 (s, 9H, Boc-CH3), 1.37 (d, J = 7.1 Hz, 3H, Ala-CH3), ^{13}C NMR (126 MHz, $CHCl_3$-*d1*) δ 173.13 (CONH), 170.29 (COOCH3), 155.66 (OCONH), 80.35 (Ctert-O), 52.48 (OCH3), 50.09 (CH), 41.28 (Gly-CH2), 28.43 (Boc-CH3), 18.41(Ala-CH3). MS calcd. for $[M+H]^+$ $C_{11}H_{21}N_2O_5^+$: 261.14; found: 261.05. Proton NMR data correspond to the literature.[2]

***N*-Boc-D-Ala-Gly-OMe (20b).** The compound was synthesized according to GOP B using Boc-D-Ala-OH (189 mg, 1.0 mmol), Gly-OMe · HCl (138 mg, 1.1 mmol), IBCF (142 µL, 1.1 mmol), NMM (132 µL, 1.2 mmol). Purification gave Boc-D-Ala-Gly-OMe (**20b**) (220 mg, 0.85 mmol, 85%) as colorless oil. ^{1}H NMR

(500 MHz, $CHCl_3$-*d1*) δ 6.74 (br-s, 1H, NH), 5.03 (br-s, 1H, NH), 4.22 (s, 1H, CH), 4.09 – 3.98 (m, 2H, Gly-CH2), 3.75 (s, 3H, OCH3), 1.44 (s, 9H, Boc-CH3), 1.37 (d, J = 7.1 Hz, 3H, Ala-CH3), ^{13}C NMR (126 MHz, $CHCl_3$-*d1*) δ 173.31 (CONH), 170.53 (COOCH3), 155.92 (OCONH), 80.67 (Ctert-O), 52.76 (OCH3), 50.38 (CH), 41.56 (Gly-CH2), 28.70 (Boc-CH3), 18.63 (Ala-CH3). ESI-MS $[C_{11}H_{20}N_2O_5+H]^+$
calcd.: 260.1 found: 261.0.

***N*-Boc-L-Ala-Sar-OEt (20c).** The compound was synthesized according to GOP A using Boc-L-Ala-OH (206 mg, 1.09 mmol), Sar-OEt · HCl (150 mg, 0.99 mmol), PyBOP (567 mg, 1.09 mmol), DIPEA (407 µL, 2.37 mmol). Purification gave the title compound **20c** (256 mg, 0.89 mmol, 89%) as colorless oil. ^{1}H NMR (500 MHz, MeOH-*d*$_4$) Major isomer: δ 4.62 (q, J = 7.0 Hz, 1H, Ala-CH), 4.32 (d, J = 17.3 Hz, 1H, 1H of Sar-CH2), 4.21 – 4.14 (m, 2H, OCH2CH3), 3.90 (d, J = 17.3 Hz, 1H, 1H of Sar-CH2), 3.18 (s, 3H, Sar-CH3), 1.44 (s, 9H, Boc-CH3), 1.29 (d, J = 7.1 Hz, 3H, Ala-CH3), 1.26 (t, J = 7.2 Hz, 3H, OCH2CH3); Minor isomer: δ 4.43 (q, J = 6.8 Hz, 1H, Ala-CH), 2.95 (s, 3H, Sar-CH3). ^{13}C NMR (126 MHz, MeOH-*d*$_4$) Major isomer: δ 175.85 (CONH), 170.61 (COOEt), 157.47 (OCONH), 80.52 (Ctert-O), 62.24 (OCH2CH3), 50.98 (Sar-CH2), 47.58 (Ala-CH), 36.87 (Sar-CH3), 28.70 (Boc-CH3), 17.69 (Ala-CH3), 14.46 (OCH2CH3); Minor isomer: δ 47.33 (Ala-CH), 35.69 (Sar-CH3). MS calcd. for $[M+H]^+$ $C_{13}H_{25}N_2O_5^+$: 289.18; found: 289.06.

***N*-Boc-D-Ala-Sar-OEt (20d).** The compound was synthesized according to GOP A using Boc-D-Ala-OH (206 mg, 1.09 mmol), Sar-OEt · HCl (150 mg, 0.99 mmol), PyBOP (567 mg, 1.09 mmol), DIPEA (407 µL, 2.37 mmol). Purification gave the title compound **20d** (222 mg, 0.77 mmol, 78%) as colorless oil. ^{1}H NMR (500 MHz, $CHCl_3$-*d1*) Major isomer: δ 5.44 (br-d, J = 8.1 Hz, 1H, NH), 4.68 (p, J = 7.0 Hz, 1H, Ala-CH), 4.32 (d, J = 17.3 Hz, 1H, 1H of Sar-CH2), 4.18 (q, J = 7.2 Hz, 2H, OCH2CH3), 3.87 (d, J = 17.3 Hz, 1H, 1H of Sar-CH2), 3.12 (s, 3H, Sar-CH3), 1.43 (s, 9H, Boc-CH3), 1.34 (d, J = 6.9 Hz, 3H, Ala-CH3), 1.27 (t, J = 7.1 Hz, 3H, OCH2CH3); Minor isomer: δ 5.33 (d, J = 8.2 Hz, 1H, NH), 4.48 (p, J = 7.0 Hz, 1H, Ala-CH), 2.98 (s, 3H, Sar-CH3), 1.42 (s, 9H, Boc-CH3). ^{13}C NMR (126 MHz, $CHCl_3$-*d1*) Major isomer: δ 173.66 (CONH), 168.97 (COOEt), 155.23 (OCONH), 79.70 (Ctert-O), 61.43 (OCH2CH3), 49.84 (Sar-CH2), 46.42 (Ala-CH), 36.43 (Sar-CH3), 28.51 (Boc-CH3), 18.94 (Ala-CH3), 14.28 (OCH2CH3); Minor isomer: δ , 46.08 (Ala-CH), 35.30 (Sar-CH3), 28.49 (Boc-CH3). MS calcd. for $[M+H]^+$ $C_{13}H_{25}N_2O_5^+$: 289.18; found: 288.99.

***N*-L-Ala-Gly-OMe * HCl (21a).** The compound was synthesized according to GOP C using Boc-L-Ala-Gly-OMe (194 mg, 0.75 mmol) and 4 M HCl/dioxane (2.5 mL, 10.0 mmol). The solvent was removed under reduced pressure to give **21a** (147 mg, 0.75 mmol, quant.) as colorless oil. ^{1}H NMR (500 MHz, MeOH-*d*$_4$) δ 4.07 – 3.95 (m, 3H, Gly-CH2 and CH), 3.74 (s, 3H, OCH3), 1.54 (d, J = 7.1 Hz, 3H, Ala-CH3), ^{13}C NMR (126 MHz, MeOH-*d*$_4$) δ 171.55 (COOCH3), 171.43 (CONH), 52.72 (OCH3), 50.18 (CH), 41.81 (Gly-CH2), 17.52 (Ala-CH3). MS calcd. for $C_6H_{13}N_2O_3^+$: 161.09; found: 161.12.

D-Ala-Gly-OMe * HCl (21b). The compound was synthesized according to GOP C using Boc-D-Ala-Gly-OMe (203 mg, 0.78 mmol) and 4 M HCl/dioxane (2.5 mL, 10.0 mmol). The solvent was removed under

reduced pressure to give **21b** (153 mg, 0.78 mmol, quant.) as colorless oil. ^{1}H NMR (500 MHz, MeOH-*d₄*) δ 4.12 – 3.93 (m, 3H, CH2-Gly and CH), 3.73 (s, 3H, OCH3), 1.54 (d, J = 7.1 Hz, 3H, Ala-CH3), ^{13}C NMR (126 MHz, MeOH-*d₄*) δ 171.72 (COOCH3), 171.60 (CONH), 52.89 (OCH3), 50.34 (CH), 41.98 (CH2-Gly), 17.69 (Ala-CH3). MS calcd. for $C_6H_{13}N_2O_3^+$: 161.09; found: 161.13.

L-Ala-Sar-OEt * HCl (21c). The compound was synthesized according to GOP C using Boc-L-Ala-Sar-OEt (236 mg, 0.82 mmol) and 4 M HCl/dioxane (3.0 mL, 12.0 mmol). The solvent was removed under reduced pressure to give **21c** (184 mg, 0.82 mmol, quant.) as colorless oil. ^{1}H NMR (500 MHz, MeOH-*d₄*) Major isomer: δ 4.49 (q, J = 7.0 Hz, 1H, Ala-CH), 4.27 (d, J = 17.3 Hz, 1H, 1H of Sar-CH2), 4.22 – 4.16 (m, 2H, OCH2CH3), 4.07 (d, J = 17.4 Hz, 1H, 1H of Sar-CH2), 3.16 (s, 3H, Sar-CH3), 1.51 (d, J = 7.0 Hz, 3H, Ala-CH3), 1.27 (t, J = 7.1 Hz, 3H, OCH2CH3); Minor isomer: δ 2.98 (s, 3H, Sar-CH3), 1.43 (d, J = 6.9 Hz, 3H, Ala-CH3). ^{13}C NMR (126 MHz, MeOH-*d₄*) Major isomer: δ 171.57 (CONH), 170.30 (COOEt), 62.48 (OCH2CH3), 50.66 (Sar-CH2), 48.16 (Ala-CH), 36.60 (Sar-CH3), 16.31 (Ala-CH3), 14.44 (OCH2CH3); Minor isomer: δ 35.38 (Sar-CH), 17.08 (Ala-CH3). MS calcd. for $C_8H_{17}N_2O_3^+$: 189.12; found: 189.13.

D-Ala-Sar-OEt * HCl (21d). The compound was synthesized according to GOP C using Boc-D-Ala-Sar-OEt (206 mg, 0.71 mmol) and 4 M HCl/dioxane (3.0 mL, 12.0 mmol). The solvent was removed under reduced pressure to give **21d** (160 mg, 0.71 mmol, quant.) as colorless oil. ^{1}H NMR (500 MHz, MeOH-*d₄*) Major isomer: δ 4.49 (q, J = 7.0 Hz, 1H, Ala-CH), 4.27 (d, J = 17.4 Hz, 1H, 1H of Sar-CH2), 4.20 (qd, J = 7.1, 1.5 Hz, 2H, OCH2CH3), 4.07 (d, J = 17.3 Hz, 1H, 1H of Sar-CH2), 3.16 (s, 3H, Sar-CH3), 1.51 (d, J = 7.0 Hz, 3H, Ala-CH3), 1.27 (t, 7.15 Hz, 3H, OCH2CH3); Minor isomer: δ 2.98 (s, 1H, Sar-CH3), 1.43 (d, J = 6.9 Hz, 1H, Ala-CH3). ^{13}C NMR (126 MHz, MeOH-*d₄*) Major isomer: δ 171.57 (CONH), 170.31 (COOEt), 62.48 (OCH2CH3), 50.67 (Sar-CH2), 48.16 (Ala-CH), 36.60 (Sar-CH3), 16.31 (Ala-CH3), 14.44 (OCH2CH3); Minor isomer: δ 35.38 (Sar-CH3), 17.08 (Ala-CH3). MS calcd. for $C_8H_{17}N_2O_3^+$: 189.12; found: 189.12.

***N*-Boc-D-Ala-L-Ala-Gly-OMe (22a).** The compound was synthesized according to GOP B using L-Ala-Gly-OMe · HCl (133 mg, 0.68 mmol), Boc-D-Ala-OH (117 mg, 0.62 mmol), IBCF (88 µL, 0.68 mmol), NMM (164 µL, 1.48 mmol). Purification by flash column chromatography gave **22a** (138 mg, 0.42 mmol, 68%) as colorless solid. ^{1}H NMR (500 MHz, $CHCl_3$-*d₁*) δ 7.28 (br-s, 1H, NH), 7.02 (br-d, J = 7.9 Hz, 1H, NH), 5.27 (br-s, 1H, NH), 4.56 (p, J = 7.2 Hz, 1H, Ala1-CH), 4.17 – 4.11 (m, 1H, Ala2-CH), 4.06 (dd, J = 18.0, 5.6 Hz, 1H, 1H of Gly-CH2), 3.93 (dd, J = 18.1, 5.0 Hz, 1H, 1H of Gly-CH2), 3.72 (s, 3H, OCH3), 1.41 (s, 9H, Boc-CH3), 1.40 (d, J = 7.1 Hz, 3H, Ala1-CH3), 1.35 (d, J = 7.1 Hz, 3H, Ala2-CH3), ^{13}C NMR (126 MHz, $CHCl_3$-*d₁*) δ 173.05 (CONH), 172.60 (CONH), 170.09 (COOCH3), 155.67 (OCONH), 80.32 (Ctert-O), 52.24 (OCH3), 50.46 (Ala2-CH), 48.70 (Ala1-CH), 41.12 (Gly-CH2), 28.24 (Boc-CH3), 18.03 (Ala2-CH3), 17.75 (Ala1-CH3). MS calcd. for $C_{14}H_{26}N_3O_6^+$: 332.18; found: 332.08.

***N*-Boc-D-Ala-D-Ala-Gly-OMe (22b).** The compound was synthesized according to GOP B using D-Ala-Gly-OMe · HCl (139 mg, 0.71 mmol), Boc-D-Ala-OH (122 mg, 0.64 mmol), IBCF (101 µL, 0.78 mmol), NMM

(188 μL, 1.7 mmol). Purification by flash column chromatography gave **22b** (164 mg, 0.49 mmol, 77%) as white solid. ^{1}H NMR (500 MHz, $CHCl_3$-*d$_1$*) δ 7.02 (br-s, 1H, NH), 6.82 (br-d, J = 7.1 Hz, 1H, NH), 5.10 (br-s, 1H, NH), 4.56 (p, J = 7.2 Hz, 1H, Ala1-CH), 4.16 (s, 1H, Ala2-CH), 4.05 (dd, J = 18.1, 5.5 Hz, 1H, 1H of Gly-CH2), 3.97 (dd, J = 18.1, 5.2 Hz, 1H, 1H of Gly-CH2), 3.73 (s, 3H, OCH3), 1.43 (s, 9H, Boc-CH3), 1.40 (d, J = 7.1 Hz, 3H, Ala1-CH3), 1.37 (d, J = 7.1 Hz, 3H, Ala2-CH3), ^{13}C NMR (126 MHz, $CHCl_3$-*d$_1$*) δ 172.88 (CONH), 172.49 (CONH), 170.17 (COOCH3), 155.89 (OCONH), 80.66 (Ctert-O), 52.46 (OCH3), 50.63 (Ala2-CH), 48.88 (Ala1-CH), 41.32 (Gly-CH2), 28.42 (Boc-CH3), 18.27 (Ala2-CH3), 18.08 (Ala1-CH3). MS calcd. for $C_{14}H_{26}N_3O_6^+$: 332.18; found: 332.08.

***N*-Boc-D-Ala-L-Ala-Sar-OEt (22c).** The compound was synthesized according to GOP B using L-Ala-Sar-OEt · HCl (179 mg, 0.80 mmol), Boc-D-Ala-OH (151 mg, 0.80 mmol), IBCF (114 μL, 0.88 mmol), NMM (192 μL, 1.76 mmol). Purification by flash column chromatography gave **22c** (208 mg, 0.58 mmol, 73%) as white solid. ^{1}H NMR (500 MHz, MeOH-*d$_4$*) Major isomer: δ 4.90 (q, J = 6.8 Hz, 1H, Ala1-CH), 4.28 (d, J = 17.3 Hz, 1H, 1H of Sar-CH2), 4.18 (q, J = 7.1 Hz, 2H, OCH2CH3), 4.11 – 4.01 (m, 1H, Ala2-CH), 3.95 (d, J = 17.3 Hz, 1H, 1H of Sar-CH2), 3.17 (s, 3H, Sar-CH3), 1.44 (s, 9H, Boc-CH3), 1.32 (d, J = 6.9 Hz, 3H, Ala1-CH3), 1.30 (d, J = 7.2 Hz, 3H, Ala2-CH3), 1.26 (t, J = 7.1 Hz, 3H, OCH2CH3); Minor isomer: δ 4.71 (q, J = 6.8 Hz, 1H, Ala1-CH), 2.95 (s, 3H, Sar-CH3). ^{13}C NMR (126 MHz, MeOH-*d$_4$*) Major isomer: δ 174.74 (CONH), 170.75 (CONH), 170.56 (COOEt), 157.54 (OCONH), 80.66 (Ctert-O), 62.30 (OCH2CH3), 51.73 (Ala2-CH), 50.90 (Sar-CH2), 46.53 (Ala1-CH), 36.91 (Sar-CH3), 28.67 (Boc-CH3), 18.30 (Ala2-CH3), 17.72 (Ala1-CH3), 14.46 (OCH2CH3); Minor isomer: δ 46.21 (Ala1-CH), 35.62 (Sar-CH3). MS calcd. for $C_{16}H_{30}N_3O_6^+$: 360.21; found: 360.51.

***N*-Boc-D-Ala-D-Ala-Sar-OEt (22d).** The compound was synthesized according to GOP B using D-Ala-Sar-OEt · HCl (160 mg, 0.71 mmol), Boc-D-Ala-OH (135 mg, 0.71 mmol), IBCF (102 μL, 0.79 mmol), NMM (172 μL, 1.58 mmol). Purification by flash column chromatography gave **22d** (190 mg, 53 mmol, 75%) as colorless oil. ^{1}H NMR (500 MHz, $CHCl_3$-*d$_1$*) Major isomer: δ 6.95 (br-d, J = 7.4 Hz, 1H, NH), 5.05 (br-s, 1H, NH), 4.96 – 4.86 (m, 1H, Ala1-CH), 4.33 – 4.27 (m, 1H, 1H of Sar-CH2), 4.18 (q, J = 7.1 Hz, 2H, OCH2CH3), 3.88 (d, J = 17.3 Hz, 1H, 1H of Sar-CH2), 3.12 (s, 3H, Sar-CH3), 1.43 (s, 9H, Boc-CH3), 1.36 (d, J = 6.8 Hz, 3H, Ala1-CH3), 1.34 (d, J = 7.1 Hz, 3H, Ala2-CH3), 1.26 (t, J = 7.2 Hz, 3H, OCH2CH3); Minor isomer: δ 4.78 – 4.66 (m, 1H, Ala1-CH), 2.98 (s, 1H, Sar-CH3). ^{13}C NMR (126 MHz, $CHCl_3$-*d$_1$*) Major isomer: δ 172.95 (CONH), 171.90 (CONH), 168.81 (COOEt), 155.35 (OCONH), 80.14 (Ctert-O), 61.49 (OCH2CH3), 50.31 (Ala2-CH), 49.84 (Sar-CH2), 45.52 (Ala1-CH), 36.44 (Sar-CH3), 28.45 (Boc-CH3), 18.93 (Ala1-CH3), 18.51 (Ala2-CH3), 14.28 (OCH2CH3); Minor isomer: δ 35.31 (Sar-CH3), 45.18 (Ala1-CH). ESI-MS $[C_{16}H_{29}N_3O_6+H]^+$ calcd.: 360.2, found: 360.1.

***N*-Boc-D-Ala-L-Ala-Gly-OH (23a).** The compound was synthesized according to GOP E using Boc-D-Ala-L-Ala-Gly-OMe (111 mg, 0.34 mmol) and 1 M NaOH (400 μL, 0.40 mmol). After workup **23a** (92 mg, 0.29 mmol, 85%) was obtained as white amorphous solid. ^{1}H NMR (500 MHz, MeOH-*d$_4$*) δ 4.42 (q, J = 7.2 Hz,

1H, Ala1-CH), 4.04 (q, J = 7.2 Hz, 1H, Ala2-CH), 3.90 (s, 2H, Gly-CH2), 1.44 (s, 9H, Boc-CH3), 1.38 (d, J = 7.2 Hz, 3H, Ala1-CH3), 1.31 (d, J = 7.2 Hz, 3H, Ala2-CH3). ^{13}C NMR (126 MHz, MeOH-d_4) δ 175.72 (COOH), 175.17 (CONH), 172.69 (CONH), 158.04 (OCONH), 80.80 (Ctert-O), 51.89 (Ala2-CH), 50.08 (Ala1-CH), 41.72 (Gly-CH2), 28.71 (Boc-CH3), 18.06 (Ala2-CH3), 18.00 (Ala1-CH3). MS calcd. for **(55a)** $C_{13}H_{24}N_3O_6^+$: 318.17; found: 318.08.

***N*-Boc-D-Ala-D-Ala-Gly-OH (23b).** The compound was synthesized according to GOP E using Boc-D-Ala-D-Ala-Gly-OMe (149 mg, 0.45 mmol) and 1 M NaOH (500 µL, 0.50 mmol). After workup **23b** (126 mg, 0.40 mmol, 88%) was obtained as white powder. ^{1}H NMR (500 MHz, MeOH-d_4) δ 4.40 (q, J = 7.4 Hz, 1H, Ala1-CH), 4.04 (q, J = 7.2 Hz, 1H, Ala2-CH), 4.01 – 3.93 (m, 1H, 1H of Gly-CH2), 3.83 (d, J = 17.8 Hz, 1H, 1H of Gly-CH2), 1.44 (s, 9H, Boc-CH3), 1.38 (d, J = 7.2 Hz, 3H, Ala1-CH3), 1.30 (d, J = 7.1 Hz, 3H, Ala2-CH2). ^{13}C NMR (126 MHz, MeOH-d_4) δ 175.92 (COOH), 175.16 (CONH), 172.68 (CONH), 157.95 (OCONH), 80.73 (Ctert-O), 51.92 (Ala2-CH), 50.19 (Ala1-CH), 41.76 (Gly-CH2), 28.71 (Boc-CH3), 17.81 (Ala2-CH3), 17.70 (Ala1-CH3). MS calcd. for $C_{13}H_{24}N_3O_6^+$: 318.17; found: 318.08.

***N*-Boc-D-Ala-L-Ala-Sar-OH (23c).** The compound was synthesized according to GOP E using Boc-D-Ala-L-Ala-Sar-OEt (191 mg, 0.53 mmol) and 1 M NaOH (585 µL, 0.59 mmol). After workup **23c** (153 mg, 0.46 mmol, 87%) was obtained as off-white amorphous solid. ^{1}H NMR (500 MHz, MeOH-d_4) Major isomer: δ 4.94 – 4.88 (m, 1H, Ala1-CH), 4.27 (d, J = 17.4 Hz, 1H, 1H of Sar-CH2), 4.10 – 4.00 (m, 1H, Ala2-CH), 3.94 (d, J = 17.4 Hz, 1H, 1H of Sar-CH2), 3.16 (s, 3H, Sar-CH3), 1.44 (s, 9H, Boc-CH3), 1.32 (d, J = 6.8 Hz, 3H, Ala1-CH3), 1.30 (d, J = 7.2 Hz, 3H, Ala2-CH3); Minor isomer: δ 2.96 (s, 3H, Sar-CH3). ^{13}C NMR (126 MHz, MeOH-d_4) Major isomer: δ 175.03 (COOH), 174.61 (CONH), 172.18 (CONH), 157.57 (OCONH), 80.69 (Ctert-O), 51.75 (Ala2-CH), 50.55 (Sar-CH2), 46.55 (Ala1-CH), 36.81 (Sar-CH3), 28.67 (Boc-CH3), 18.28 (Ala2-CH3), 17.75 (Ala1-CH3); Minor isomer: δ 35.62 (Sar-CH3). MS calcd. for $C_{14}H_{26}N_3O_6^+$: 332.18; found: 332.22.

***N*-Boc-D-Ala-D-Ala-Sar-OH (23d).** The compound was synthesized according to GOP E using Boc-D-Ala-D-Ala-Sar-OEt (175 mg, 0.49 mmol) and 1 M NaOH (536 µL, 0.53 mmol). After workup **23d** (141 mg, 0.43 mmol, 88%) was obtained as white amorphous solid. ^{1}H NMR (500 MHz, CHCl3-d_1) Major isomer: δ 7.52 (br-d, J = 7.7 Hz, 1H, NH), 5.44 (br-d, J = 7.6 Hz, 1H, NH), 5.00 (p, J = 6.9 Hz, 1H,Ala1-CH), 4.23 (d, J = 17.4 Hz, 1H, 1H of Sar-CH2), 4.00 – 3.94 (m, 1H, 1H of Sar-CH2), 3.17 (s, 3H, Sar-CH3), 1.43 (s, 9H, Boc-CH3), 1.35 (d, J = 6.9 Hz, 3H, Ala1-CH3), 1.32 (d, J = 7.1 Hz, 3H, Ala2-CH3); Minor isomer: δ 4.89 – 4.78 (m, 1H, Ala1-CH), 3.00 (s, 3H, Sar-CH3). ^{13}C NMR (126 MHz, CHCl3-d_1) Major isomer: δ 173.53 (COOH), 172.40 (CONH), 171.19 (CONH), 155.79 (OCONH), 80.43 (Ctert-O), 50.24 (Sar-CH2), 50.05 (Ala2-CH), 45.41 (Ala1-CH), 36.91 (Sar-CH3), 28.46 (Boc-CH3), 19.43 (Ala2-CH3), 18.33 (Ala1-CH3); Minor isomer: δ 45.41 (Ala1-CH), 35.49 (Sar-CH3). MS calcd. for $C_{14}H_{26}N_3O_6^+$: 332.18; found: 331.99.

***N*-Boc-D-Ala-L-Ala-Gly-SNAc (26a).** The compound was synthesized according to GOP B using Boc-D-Ala-L-Ala-Gly-OH (82 mg, 0.26 mmol), IBCF (67 µL, 0.52 mmol), *N*-acetylcysteamine (62 mg, 0.52 mmol) and NMM (57 µL, 0.52 mmol). Purification by flash column chromatography gave **26a** (17 mg, 0.04 mmol, 16%) as white solid. ^{1}H NMR (500 MHz, $CHCl_3$-*d_1*) δ 7.63 (br-s, 1H, NH), 6.87 (br-d, J = 8.1 Hz, 1H, NH), 6.30 (br-s, 1H, NH), 5.32 (br-d, J = 6.5 Hz, 1H, NH), 4.64 – 4.49 (m, 1H, Ala1-CH)), 4.21 (dd, J = 17.3, 5.9 Hz, 1H, 1H of Gly-CH2), 4.16 – 4.07 (m, 1H, Ala2-CH), 4.05 – 3.98 (m, 1H, 1H of Gly-CH2), 3.49 – 3.36 (m, 2H, CONHCH2), 3.03 (t, J = 6.2 Hz, 2H, COSCH2), 1.98 (s, 3H, SNAc-CH3), 1.43 (s, 3H, Ala2-CH), 1.42 (s, 9H, Boc-CH3), 1.38 (d, J = 7.0 Hz, 3H, Ala1-CH3). ^{13}C NMR (126 MHz, $CHCl_3$-*d_1*) δ 197.97 (COS), 173.35 (CONH), 172.90 (CONH), 171.08 (CONH), 156.14 (OCONH), 80.66 (Ctert-O), 50.84 (Ala2-CH), 49.47 (Gly-CH2), 48.98 (Ala1-CH), 39.29 (CONHCH2), 28.51 (COSCH2), 28.47 (Boc-CH3), 23.21 (SNAc-CH3), 17.87 (Ala1-CH3), 17.44 (Ala2-CH3). MS calcd. for $C_{17}H_{31}N_4O_6S$+: 419.20; found: 419.12.

***N*-Boc-D-Ala-D-Ala-Gly-SNAc (26b).** The compound was synthesized according to GOP F using Boc-D-Ala-D-Ala-Gly-OH (50 mg, 0.15 mmol), TFFH (46 mg, 0.17 mmol), *N*-acetylcysteamine (20 mg, 0.17 mmol) and DIPEA (53 µL, 0.31 mmol). Purification by flash column chromatography gave **26b** (4 mg, 0.01 mmol, 6%) as white solid. ^{1}H NMR (300 MHz, $CHCl_3$-*d_1*) δ 7.45 (br-s, 1H, NH), 6.78 (br-s, 1H, NH), 6.57 (br-s, 1H, NH), 5.03 (br-s, 1H, NH), 4.64 -4.46 (m, 1H, Ala1-CH), 4.26 – 4.00 (m, 3H, Ala2-CH and Gly-CH2), 3.51 – 3.38 (m, 2H, CONHCH2), 3.12- 3.01 (m, 2H, COSCH2), 2.03 (s, 3H, SNAc-CH3), 1.44 (s, 9H, Boc-CH3), 1.43 (d, 3H, Ala2-CH3), 1.40 (d, J = 7.4 Hz, 3H, Ala1-CH3).

***N*-Boc-D-Ala-L-Ala-Sar-SNAc (26c).** The compound was synthesized according to GOP F using Boc-D-Ala-L-Ala-Sar-OH (50 mg, 0.15 mmol), TFFH (44 mg, 0.17 mmol), *N*-acetylcysteamine (20 mg, 0.17 mmol) and DIPEA (52 µL, 0.30 mmol). Purification by flash column chromatography gave **26c** (36 mg, 0.08 mmol, 53%) as white solid. ^{1}H NMR (500 MHz, DMSO-*d_6*) Major isomer: δ 8.03 (br-d, J = 6.1 Hz, 1H, NH), 7.90 (br-d, J = 7.7 Hz, 1H, NH), 6.92 (br-d, J = 7.7 Hz, 1H, NH), 4.81 – 4.74 (m, 1H, Ala1-CH), 4.42 (d, J = 17.1 Hz, 1H, 1H of Sar-CH2), 4.11 (d, J = 17.0 Hz, 1H, 1H of Sar-CH2), 4.02 – 3.91 (m, 1H, Ala2-CH), 3.21 – 3.12 (m, 2H, CONHCH2), 3.10 (s, 3H, Sar-CH3), 2.93 – 2.88 (m, 2H, COSCH2), 1.78 (s, 3H, SNAc-CH3), 1.37 (s, 9H, Boc-CH3), 1.20 (d, J = 6.8 Hz, 3H, Ala2-CH3), 1.15 (d, J = 7.2 Hz, 3H, Ala1-CH3); Minor isomer: δ 4.57 – 4.50 (m, 1H, Ala1-CH), 2.86 (s, 3H, Sar-CH3). ^{13}C NMR (126 MHz, DMSO-*d_6*) Major isomer: δ 196.63 (COS), 172.44 (CONH), 171.99 (CONH), 169.27 (CONH), 154.96 (OCONH), 78.08 (Ctert-O), 57.46 (Sar-CH2), 49.66 (Ala2-CH), 44.36 (Ala1-CH), 38.10 (CONHCH2), 36.30 (Sar-CH3), 28.16 (Boc-CH3), 27.51 (COSCH2), 22.50 (SNAc-CH3), 18.19 (Ala1-CH3), 17.42 (Ala2-CH3); Minor isomer: δ 44.12 (Ala1-CH), 35.06 (Sar-CH3). MS calcd. for $C_{18}H_{33}N_4O_6S^+$: 433.21; found: 433.76.

***N*-Boc-D-Ala-D-Ala-Sar-SNAc (26d).** The compound was synthesized according to GOP F using Boc-D-Ala-D-Ala-Sar-OH (40 mg, 0.12 mmol), TFFH (36 mg, 0.17 mmol), *N*-acetylcysteamine (16 mg, 0.13 mmol) and DIPEA (41 µL, 0.24 mmol). Purification by flash column chromatography gave **26d** (23 mg, 0.05 mmol, 42%) as white solid. ^{1}H NMR (300 MHz, $CHCl_3$-*d_1*) Major isomer: δ 7.00 (br-d, J = 7.8 Hz, 1H, NH), 6.47 (br-s,

1H, NH), 5.03 (br-s, 1H, NH), 4.98 -4.88 (m, 1H, Ala1-CH), 4.39 (d, J = 16.8 Hz, 1H, 1H of Sar-CH2), 4.20 – 4.02 (m, 2H, 1H of Sar-CH2 and Ala1-CH), 3.47 (br-s, 2H, CONHCH2), 3.18 (s, 3H, Sar-CH3), 3.12 – 3.05 (m, 1H, COSCH2), 2.04 (s, 3H, SNAc-CH3), 1.44 (s, 9H, Boc-CH3), 1.39 (d, J = 6.8 Hz, 3H, Ala2-CH3), 1.36 (d, J = 7.1 Hz, 3H, Ala1-CH3). MS calcd. for $C_{18}H_{33}N_4O_6S^+$: 433.21; found: 433.00.

D-Ala-L-Ala-Gly-SNAc * HCl (9). The compound was synthesized according to GOP C using Boc-D-Ala-L-Ala-Gly-SNAc (4 mg, 9.6 µmol) in 4 M HCl in dioxane (100 µL, 0.4 mmol). The solvent was removed under reduced pressure to give **9** (3.3 mg, 9.6 µmol, quant.) as yellowish solid. MS calcd. for $C_{12}H_{23}N_4O_4S^+$: 319.14; found: 319.06. HR-MS [$C_{12}H_{22}N_4O_4S$ + H]$^+$ calcd. 319.1435, found 319.1427.

D-Ala-D-Ala-Gly-SNAc * HCl (10). The compound was synthesized according to GOP C using Boc-D-Ala-D-Ala-Gly-SNAc (7 mg, 17 µmol) in 4 M HCl in dioxane (200 µL, 0.8 mmol). The solvent was removed under reduced pressure to give **10** (5 mg, quant.) as yellowish solid. MS calcd. for $C_{12}H_{23}N_4O_4S^+$: 319.14; found: 319.18. HR-MS [$C_{12}H_{22}N_4O_4S$ + H]$^+$ calcd. 319.1435, found 319.1427.

D-Ala-L-Ala-Sar-SNAc * HCl (11). The compound was synthesized according to GOP C using Boc-D-Ala-L-Ala-Sar-SNAc (10 mg, 23 µmol) in 4 M HCl in dioxane (200 µL, 0.8 mmol). The solvent was removed under reduced pressure to give **11** (8.5 mg, 23 µmol, quant.) as yellowish solid. MS calcd. for $C_{13}H_{25}N_4O_4S^+$: 333.16; found: 333.07. HR-MS [$C_{13}H_{24}N_4O_4S$ + H]$^+$ calcd. 333.1591, found 333.1582.

D-Ala-D-Ala-Sar-SNAc * HCl (12). The compound was synthesized according to GOP C using Boc-D-Ala-D-Ala-Sar-SNAc (6 mg, 14 µmol) in 4 M HCl in dioxane (100 µL, 0.4 mmol). The solvent was removed under reduced pressure to give **12** (5 mg, 14 µmol, quant.) as yellowish solid. MS calcd. for $C_{13}H_{25}N_4O_4S^+$: 333.16; found: 333.37. HR-MS [$C_{13}H_{24}N_4O_4S$ + H]$^+$ calcd. 333.1591, found 333.1583.

***N*-Boc-L-Ser(OBn)-Sar-OEt (30a).** The compound was synthesized according to GOP A using Boc-L-Ser(OBn)-OH (584 mg, 1.98 mmol), Sar-OEt · HCl (303 mg, 2.00 mmol), PyBOP (1.24 g, 2.4 mmol) and DIPEA (822 µL, 4.8 mmol). Purification by flash column chromatography gave **30a** (688 mg, 1.74 mmol, 88%) as colorless oil. ^{1}H NMR (500 MHz, MeOH-d_4) Major isomer: δ 7.39 – 7.24 (m, 5H, H-Ph), 4.87 (t, J = 6.3 Hz, 1H, Ser-CH), 4.58 – 4.51 (m, 2H, benzyl-CH2), 4.22 – 4.18 (m, 1H, 1H of Sar-CH2), 4.18 – 4.13 (m, 2H, OCH2CH3), 4.03 (d, J = 17.3 Hz, 1H, 1H of Sar-CH2), 3.69 (dd, J = 9.8, 6.1 Hz, 1H, 1H of Ser-CH2), 3.65 – 3.55 (m, 1H, 1H of Ser-CH2), 3.17 (s, 3H, Sar-CH3), 1.44 (s, 9H, Boc-CH3), 1.24 (t, J = 7.2 Hz, 3H, OCH2CH3); Minor isomer: δ 4.66 (t, J = 6.6 Hz, 1H, Ser-CH), 2.96 (s, 3H, Sar-CH3). ^{13}C NMR (126 MHz, MeOH-d_4) Major isomer: δ 173.27 (CONH), 170.47 (COOEt), 157.54 (OCONH), 139.34 (C-Ph), 129.36 (C-Ph), 128.81 (C-Ph), 128.77 (C-Ph), 128.72 (C-Ph), 80.76 (Ctert-O), 74.21 (benzyl-CH2), 70.92 (Ser-CH2), 62.24 (OCH2CH3), 51.76 (Ser-CH), 51.01 (Sar-CH2), 37.11 (Sar-CH3), 28.68 (Boc-CH3), 14.46 (OCH2CH3); Minor isomer: δ 52.28 (Ser-CH), 35.67 (Sar-CH3). MS calcd. for $C_{20}H_{31}N_2O_6^+$: 395.22; found: 395.59.

***N*-Boc-L-Ser(OBn)-Gly-OMe (30b).** The compound was synthesized according to GOP B using Boc-L-Ser(OBn)-OH (354 mg, 1.2 mmol), Gly-OMe · HCl (150 mg, 1.2 mmol), IBCF (155 µL, 1.2 mmol) and NMM (264 µL, 2.4 mmol). Purification by flash column chromatography gave **30b** (373 mg, 1.02 mmol, 85%) as colorless oil. ^{1}H NMR (500 MHz, MeOH-*d$_4$*) δ 7.42 – 7.18 (m, 5H, H-Ph), 4.59 – 4.50 (m, 2H, benzyl-CH2), 4.34 (s, 1H, Ser-CH), 4.01 – 3.91 (m, 2H, Ser-CH2), 3.71 (s, 5H, OCH3 and Gly-CH2), 1.45 (s, 9H, Boc-CH3). ^{13}C NMR (126 MHz, MeOH-*d$_4$*) δ 173.71 (CONH), 171.80 (COOCH3), 158.07 (OCONH), 139.64 (C-Ph), 129.70 (C-Ph), 129.19 (C-Ph), 129.06 (C-Ph), 81.27 (Ctert-O), 74.49 (benzyl-CH2), 71.35 (Ser-CH2), 56.32 (Ser-CH), 52.93 (OCH3), 42.29 (Gly-CH2), 29.01 (Boc-CH3). MS calcd. for $C_{18}H_{27}N_2O_6^+$: 367.19; found: 367.52. NMR data correspond to the literature [3].

L-Ser(OBn)-Sar-OEt * HCl (31a). The compound was synthesized according to GOP C using Boc-L-Ser(OBn)-Sar-OEt (668 mg, 1.69 mmol) and 4 M HCl in dioxane (5 mL, 20.0 mmol). The solvent was removed under reduced pressure to give **31a** (560 mg, 1.69 mmol, quant.) as colorless oil. MS calcd. for $[C_{15}H_{22}N_2O_4+H]^+$: 295.17; found: 295.17.

L-Ser(OBn)-Gly-OMe * HCl (31b). The compound was synthesized according to GOP C using Boc-L-Ser(OBn)-Gly-OMe (373 mg, 1.02 mmol) and 4 M HCl in dioxane (3 mL, 12.0 mmol). The solvent was removed under reduced pressure to give **31b** (308 mg, 1.02 mmol, quant.) as colorless oil. ^{1}H NMR (500 MHz, MeOH-*d$_4$*) δ 7.44 – 7.24 (m, 5H, H-Ph), 4.68 – 4.57 (m, 2H, benzyl-CH2), 4.18 (dd, J = 6.6, 3.9 Hz, 1H, Ser-CH), 4.01 (d, J = 3.0 Hz, 2H, Gly-CH2), 3.91 (dd, J = 10.6, 3.9 Hz, 1H, 1H of Ser-CH2), 3.80 (dd, J = 10.6, 6.5 Hz, 1H, 1H of Ser-CH2), 3.72 (s, 3H, OCH3). ^{13}C NMR (126 MHz, MeOH-*d$_4$*) δ 171.29 (CONH), 168.38 (COOCH3), 138.52 (C-Ph), 129.50 (C-Ph), 129.13 (C-Ph), 129.06 (C-Ph), 74.56 (benzyl-CH2), 69.03 (Ser-CH2), 54.55 (Ser-CH), 52.73 (OCH3), 41.90 (Gly-CH2). MS calcd. for $C_{13}H_{19}N_2O_4^+$: 267.13; found: 266.92.

***N*-Boc-D-Ala-L-Ser(OBn)-Sar-OEt (32a).** The compound was synthesized according to GOP B using L-Ser(OBn)-Sar-OEt · HCl (560 mg, 1.69 mmol), Boc-D-Ala-OH (314 mg, 1.66 mmol), IBCF (241 µL, 1.86 mmol) and NMM (408 µL, 3.72 mmol). Purification by flash column chromatography gave **32a** (680 mg, 1.46 mmol, 88%) as colorless solid. ^{1}H NMR (500 MHz, MeOH-*d$_4$*) Major isomer: δ 7.39 – 7.20 (m, 5H, H-Ph), 5.15 (t, J = 5.9 Hz, 1H, Ser-CH), 4.56 -4.53 (m, 2H, benzyl-CH2), 4.20 (d, J = 15.0 Hz, 1H, 1H of Sar-CH2), 4.18 – 4.14 (m, 2H, OCH2CH3), 4.10 (q, J = 7.1 Hz, 1H, Ala-CH), 4.04 (d, J = 17.2 Hz, 1H, 1H of Sar-CH2), 3.71 (dd, J = 9.9, 5.9 Hz, 1H, 1H of Ser-CH2), 3.68 – 3.59 (m, 1H, 1H of Ser-CH2), 3.17 (s, 3H, Sar-CH3), 1.42 (s, 9H, Boc-CH3), 1.29 (d, J = 7.2 Hz, 3H, Ala-CH3), 1.25 (t, J = 7.1 Hz, 3H, OCH2CH3); Minor isomer: δ 4.95 (t, J = 6.4 Hz, 1H, Ser-CH), 2.96 (s, 3H, Sar-CH3). ^{13}C NMR (126 MHz, MeOH-*d$_4$*) Major isomer: δ 175.41 (CONH), 172.32 (CONH), 170.45 (COOEt), 157.55 (OCONH), 139.26 (C-Ph), 129.38 (C-Ph), 128.84 (C-Ph), 128.80 (C-Ph), 128.74 (C-Ph), 80.66 (Ctert-O), 74.26 (benzyl-CH2), 70.57 (Ser-CH2), 62.29 (OCH2CH3), 51.69 (Ala-CH), 50.98 (Sar-CH2), 50.54 (Ser-CH), 37.16 (Sar-CH3), 28.68 (Boc-CH3), 18.35

(Ala-CH3), 14.47 (OCH2CH3); Minor isomer: δ 50.19 (Ser-CH), 35.65 (Sar-CH3). MS calcd. for $C_{23}H_{36}N_3O_7^+$: 466.25; found: 466.21.

***N*-Boc-D-Ala-L-Ser(OBn)-Gly-OMe (32b).** The compound was synthesized according to GOP B using L-Ser(OBn)-Gly-OMe · HCl (308 mg, 1.0 mmol), Boc-D-Ala-OH (189 mg, 1.0 mmol), IBCF (144 µL, 1.1 mmol) and NMM (244 µL, 2.2 mmol). Purification by flash column chromatography gave **32b** (353 mg, 0.81 mmol, 81%) as colorless solid. ^{1}H NMR (500 MHz, MeOH-d_4 δ 7.40 – 7.22 (m, 5H, H-Ph), 4.61 (t, J = 4.9 Hz, 1H, Ser-CH), 4.55 – 4.54 (m, 2H, benzyl-CH2), 4.13 – 4.06 (m, 1H, Ala-CH), 4.01 (d, J = 17.6 Hz, 1H, 1H of Gly-CH2), 3.91 (d, J = 17.5 Hz, 1H, 1H of Gly-CH2), 3.85 (dd, J = 9.8, 5.5 Hz, 1H, 1H of Ser-CH2), 3.74 (dd, J = 9.9, 4.2 Hz, 1H, 1H of Ser-CH2), 3.70 (s, 3H, OCH3), 1.41 (s, 9H, Boc-CH3), 1.30 (d, J = 7.1 Hz, 3H, Ala-CH3). ^{13}C NMR (126 MHz, MeOH-d_4) δ 176.22 (CONH), 172.52 (CONH), 171.35 (COOCH3), 157.98 (OCONH), 139.27 (C-Ph), 129.36 (C-Ph), 128.85 (C-Ph), 128.72 (C-Ph), 80.77 (Ctert-O), 74.21 (benzyl-CH2), 70.47 (Ser-CH2), 54.69 (Ser-CH), 52.60 (OCH3), 51.83 (Ala-CH), 42.00 (Gly-CH2), 28.71 (Boc-CH3), 17.72 (Ala-CH3). MS calcd. for $C_{21}H_{32}N_3O_7^+$: 438.22; found: 438.09.

***N*-Boc-D-Ala-L-Ser(OH)-Sar-OEt (33a).** The compound was synthesized according to GOP D using Boc-D-Ala-L-Ser(OBn)-Sar-OEt (680 mg, 1.46 mmol) and Pd/C (68 mg, 10 wt %). After filtration the solvent was evaporated to give **33a** (533 mg, 1.42 mmol, 97%) as white amorphous solid. ^{1}H NMR (500 MHz, CHCl$_3$-d_1) Major isomer: δ 5.13 – 4.98 (m, 1H, Ser-CH), 4.34 (d, J = 17.3 Hz, 1H, 1H of Sar-CH2), 4.20 (q, J = 7.2 Hz, 3H, OCH2CH3 and Ala-CH), 3.88 (d, J = 17.3 Hz, 1H, 1H of Sar-CH2), 3.82 (d, J = 4.8 Hz, 2H, Ser-CH2), 3.18 (s, 3H, Sar-CH3), 2.70 (br-s, 1H, OH), 1.43 (s, 9H, Boc-CH3), 1.37 (d, J = 7.1 Hz, 3H, Ala-CH3), 1.27 (t, J = 7.1 Hz, 3H, OCH2CH3); Minor isomer: δ 4.73 (dt, J = 8.3, 4.3 Hz, 1H, Ser-CH), 2.97 (s, 3H, Sar-CH3). ^{13}C NMR (126 MHz, CHCl$_3$-d_1) Major isomer: δ 173.02 (COOH), 171.16 (CONH), 169.08 (CONH), 155.62 (OCONH), 80.59 (Ctert-O), 63.77 (Ser-CH2), 61.81 (OCH2CH3), 51.63 (Ser-CH), 51.54 (Ala-CH), 50.22 (Sar-CH2), 36.94 (Sar-CH3), 28.41 (Boc-CH3), 18.62 (Ala-CH3), 14.26 (OCH2CH3); Minor isomer: δ 50.74 (Ser-CH), 35.35 (Sar-CH3). MS calcd. for $C_{16}H_{30}N_3O_7^+$: 376.21; found: 376.03.

***N*-Boc-D-Ala-L-Ser(OH)-Gly-OMe (33b).** The compound was synthesized according to GOP D using Boc-D-Ala-L-Ser(OBn)-Gly-OMe (330 mg, 0.75 mmol) and Pd/C (33 mg, 10 wt %). After filtration the solvent was evaporated to give **33b** (254 mg, 0.73 mmol, 97%). ^{1}H NMR (500 MHz, MeOH-d_4) δ 4.45 (t, J = 5.0 Hz, 1H, Ser-CH), 4.08 (q, J = 7.1 Hz, 1H, Ala-CH), 4.02 (d, J = 17.6 Hz, 1H, 1H of Gly-CH2), 3.92 (d, J = 17.6 Hz, 1H, 1H of Gly-CH2), 3.89 – 3.83 (m, 1H, 1H of Ser-CH2), 3.81 (dd, J = 11.1, 4.6 Hz, 1H, 1H of Ser-CH2), 3.72 (s, 3H, OCH3), 1.43 (s, 9H, Boc-CH3), 1.32 (d, J = 7.1 Hz, 3H, Ala-CH3). ^{13}C NMR (126 MHz, MeOH-d_4) δ 176.29 (CONH), 172.81 (CONH), 171.58 (COOCH3), 158.05 (OCONH), 80.80 (Ctert-O), 62.75 (Ser-CH2), 56.70 (Ser-CH), 52.65 (OCH3), 51.96 (Ala-CH), 41.96 (Gly-CH2), 28.71 (Boc-CH3), 17.66 (Ala-CH3). MS calcd. for $C_{14}H_{26}N_3O_7^+$: 348.18; found: 348.65.

***N*-Boc-D-Ala-Dha-Sar-OEt (34a).** Boc-D-Ala-L-Ser(OH)-Sar-OEt (**33a**) (143 mg, 0.38 mmol) was dissolved in CH_2Cl_2 and the solution was degassed by freeze-thawing cycles in vacuo. To the solution was added CuCl (3.7 mg, 10 mol %), EDC hydrochloride (145 mg, 0.76 mmol) and DBU (57 µL, 0.38 mmol) and the reaction mixture was stirred at room temperature overnight. The solvent was removed under reduced pressure and purification by flash column chromatography (linear gradient of PE/EtOAc 5–100%) gave **34a** (96 mg, 0.27 mmol, 71%) as colorless solid. ^1H NMR (500 MHz, $CHCl_3$-d_1) Major isomer: δ 9.66 (br-s, 1H, NH), 7.02 (br-d, J = 9.1 Hz, 1H, NH), 5.41 (s, 1H, 1H of olefin-CH2), 4.66 (s, 1H, 1H of olefin-CH2), 4.17 – 4.07 (m, 4H, Sar-CH2 and OCH2CH3), 4.07 – 3.93 (m, 1H, Ala-CH), 2.99 (s, 3H, Sar-CH3), 1.37 (s, 9H, Boc-CH3), 1.23 (d, J = 7.1 Hz, 3H, Ala-CH3), 1.21 – 1.16 (m, 3H, OCH2CH3); Minor isomer: δ 5.25 (s, 1H, 1H of olefin-CH2), 4.50 (s, 1H, 1H of olefin-CH2), 2.87 (s, 3H, Sar-CH3). ^{13}C NMR (126 MHz, $CHCl_3$-d_1) Major isomer: δ 171.70 (CONH), 168.87 (COOEt), 166.92 (Colefin-CONH), 155.09 (OCONH), 137.07 (Colefin), 101.34 (olefin-CH2), 78.10 (Ctert-O), 60.51 (OCH2CH3), 49.80 (Ala-CH), 48.45 (Sar-CH2), 37.50 (Sar-CH3), 28.18 (Boc-CH3), 17.79 (Ala-CH3), 14.04 (OCH2CH3); Minor isomer: δ 100.36 (olefin-CH2), 33.55 (Sar-CH3). MS calcd. for $C_{16}H_{28}N_3O_6^+$: 358.20; found: 358.09.

***N*-Boc-D-Ala-Dha-Gly-OMe (34b).** Boc-D-Ala-L-Ser(OH)-Gly-OMe **33b** (50 mg, 0.14 mmol) was dissolved in CH_2Cl_2 and the solution was degassed by freeze-thawing cycles in vacuo. To the solution was added CuCl (1.4 mg, 10 mol %) and EDC hydrochloride (55 mg, 0.29 mmol) and the reaction mixture was stirred at room temperature overnight. The solvent was removed under reduced pressure and purification by flash column chromatography (linear gradient of PE/EtOAc 5–100%) gave **34b** (35 mg, 0.11 mmol, 79%) as white solid. ^1H NMR (300 MHz, $CHCl_3$-d_1) δ 8.56 (br-s, 1H, NH), 6.72 (br-s, 1H, NH), 6.49 (s, 1H, 1H of olefin-CH2), 5.36 (s, 1H, 1H of olefin-CH2), 5.01 (br-s, 1H, NH), 4.24 (br-s, 1H, Ala-CH), 4.11 (d, J = 5.1 Hz, 2H, Gly-CH2), 3.79 (s, 3H, OCH3), 1.44 (s, 9H, Boc-CH3), 1.39 (d, J = 7.0 Hz, 3H, Ala-CH3). ^{13}C NMR (126 MHz, $CHCl_3$-d_1) δ 171.86 (CONH), 170.06 (COOCH3), 164.01 (Colefin-CONH), 155.70 (OCONH), 133.78 (Colefin), 103.07 (olefin-CH2), 80.50 (Ctert-O), 52.73 (OCH3), 52.72 (Ala-CH), 41.83 (Gly-CH2), 28.42 (Boc-CH3), 18.48 (Ala-CH3). MS calcd. for $C_{14}H_{24}N_3O_6^+$: 330.17; found: 330.07.

***N*-Boc-D-Ala-Dha-Gly-SNAc (35b).** Boc-D-Ala-Dha-Gly-OMe (**34b**) (33 mg, 0.10 mmol) was dissolved in 1.75 mL of THF/MeOH/H_2O (4/1/2) and cooled to 0 °C. Aqueous 0.5 N LiOH (219 µL, 0.11 mmol) was added and the reaction mixture was stirred for 30 min. Then, the reaction was diluted with CH_2Cl_2 (10 mL) and the organic layer was washed with $KHSO_4$ (1 × 8 mL) and subsequently the aqueous layer was reextracted with CH_2Cl_2 (2 × 10 mL). The combined organic layers were dried over Na_2SO_4, filtered and the solvent was removed under reduced pressure. The crude product was directly converted according to GOP F using Boc-D-Ala-Dha-Gly-OH (31 mg, 0.10 mmol), TFFH (29 mg,0.11 mmol), *N*-acetylcysteamine (13 mg, 0.11 mmol) and DIPEA (34 µL, 0.20 mmol). Purification by flash column chromatography gave **35b** (12 mg, 0.03 mmol, 30%) as yellowish solid. ^1H NMR (500 MHz, DMSO-d_6) δ 9.13 (br-t, J = 5.9 Hz, 1H, NH), 9.05 (br-s, 1H, NH), 8.03 (br-t, J = 5.7 Hz, 1H, NH), 7.36 (br-d, J = 7.1 Hz, 1H, NH), 6.29 (s, 1H, 1H of olefin-CH2), 5.58 (s, 1H, 1H of olefin-CH2), 4.14 – 3.98 (m, 3H, Gly-CH2 and Ala-CH), 3.21 – 3.08 (m, 2H, CONHCH2), 2.90

(dd, J = 7.6, 6.4 Hz, 2H, COSCH2), 1.78 (s, 3H, SNAc-CH3), 1.38 (s, 9H, Boc-CH3), 1.20 (d, J = 7.2 Hz, 3H, Ala-CH3). ^{13}C NMR (126 MHz, DMSO-d_6) δ 197.65 (COS), 172.30 (CONH), 169.24 (CONH), 164.19 (CONH), 155.28 (OCONH), 134.27 (olefin-C), 102.85 (olefin-CH2), 78.46 (Ctert-O), 50.69 (Ala-CH), 49.34 (Gly-CH2), 38.11 (CONHCH2), 28.14 (Boc-CH3), 27.52 (COSCH2), 22.51 (SNAc-CH3), 17.32 (Ala-CH3). MS calcd. for $[C_{17}H_{28}N_4O_6S+Na]^+$: 439.16; found: 439.18.

D-Ala-Dha-Gly-SNAc * HCl (13). The compound was synthesized according to GOP C using Boc-D-Ala-Dha-Gly-SNAc (12 mg, 29 µmol) and 4 M HCl in dioxane (200 µL, 0.8 mmol). The solvent was removed under reduced pressure to give **13** (10 mg, 29 µmol, quant.) as yellowish solid. MS calcd. for $[C_{12}H_{21}N_4O_4S]^+$: 317.13; found: 317.02. HR-MS $[C_{12}H_{20}N_4O_4S + H]^+$ calcd. 317.1278, found 317.1272.

L-Ser(OBn)-OEt * HCl (37). *N*-Boc-*O*-benzyl-L-serine (2.00 g; 6.76 mmol) was dissolved in EtOH (80 mL) and thionyl chloride (1.47 mL; 20.3 mmol) was added drop-wise to the solution. After stirring for 4 h at 78 °C the solvent was removed in vacuo to give analytically pure **37** (1.75 g; quant.) as colorless solid. ^{1}H NMR (500 MHz, DMSO-d_6) δ 8.88 (bs, 3H, N$\underline{H}_3$Cl), 7.45 – 7.23 (m, 5H, ArH), 4.59 (d, *J* = 12.3 Hz, 1H, C$\underline{H}_2$Ph), 4.46 (d, *J* = 12.2 Hz, 1H, C$\underline{H}_2$Ph), 4.28 (t, *J* = 3.2 Hz, 1H, Hα-Ser), 4.24 – 4.11 (m, 2H, C$\underline{H}_2$CH$_3$), 3.91 (ddt, *J* = 10.4, 3.8, 1.9 Hz, 1H, Hβ-Ser), 3.85 (dd, *J* = 10.4, 3.7 Hz, 1H, Hβ-Ser), 1.18 (t, *J* = 7.1 Hz, 3H, CH$_2$C$\underline{H}_3$). ^{13}C NMR (126 MHz, DMSO-d_6) δ 167.68 (CO), 137.45 (C-Ar), 128.25 (2x$\underline{C}$H-Ar), 127.68 ($\underline{C}$H-Ar), 127.63 (2x$\underline{C}$H-Ar), 72.38 ($\underline{C}$H$_2$Ph), 67.26 (Cβ-Ser), 61.81 ($\underline{C}$H$_2$CH$_3$), 52.37 (Cα-Ser), 13.93 (CH$_2\underline{C}$H$_3$). ESI-MS $[C_{12}H_{17}NO_3 + H]^+$ calcd 224.1, found 224.1

***N*-Boc-D-Ala-L-Ser(OBn)-OEt (38).** *N*-Boc-D-alanine (1.25 g; 6.60 mmol) was dissolved in anhydrous THF (66 mL) and stirred for 10 min at −20 °C. *N*-Methylmorpholine (1.74 mL; 14.5 mmol) and isobutyl chloroformate (0.95 mL; 7.26 mmol) was added drop-wise over 10 min at −20 °C and the reaction was stirred for further 20 min at −20 °C. A solution of *O*-benzyl-L-serine ethyl ester hydrochloride (**37**) (1.71 g; 6.6 mmol) in 2 mL anhydrous THF was added dropwise over 30 min and the reaction mixture was stirred for 1 h at −20 °C. The reaction mixture was diluted with EtOAc (70 mL) and washed with satd. $NaHSO_3$ solution (20 mL), satd. $NaHCO_3$ solution (20 mL) and brine (20 mL). The organic layer was dried over Na_2SO_4, filtered and the volatiles were removed in vacuo. Purification by flash column chromatography (SiO_2; PE to PE/EtOAc = 2:1) yielded benzyl protected ethyl ester **38** (2.37 g; 6.00 mmol; 91%) as a colorless solid. ^{1}H NMR (500 MHz, DMSO-d_6) δ 8.10 (d, *J* = 8.1 Hz, 1H, N$\underline{H}$-Ser), 7.39 – 7.24 (m, 5H, ArH), 7.00 (d, *J* = 7.9 Hz, 1H, N$\underline{H}$-Ala), 4.57 – 4.42 (m, 3H, C$\underline{H}_2$Ph, Hα-Ser), 4.15 – 4.01 (m, 3H, C$\underline{H}_2$CH$_3$, Hα-Ala), 3.74 (dd, *J* = 9.8, 5.0 Hz, 1H, Hβ-Ser), 3.61 (dd, *J* = 9.7, 4.2 Hz, 1H, Hβ-Ser), 1.37 (s, 9H, C(C$\underline{H}_3$)$_3$), 1.20 – 1.09 (m, 6H, CH$_2$C$\underline{H}_3$, Hβ-Ala). ^{13}C NMR (126 MHz, DMSO-d_6) δ 172.95 (CO), 169.90 (CO), 155.02 (CO), 137.87 (C-Ar), 128.21 (2x$\underline{C}$H-Ar), 127.52 ($\underline{C}$H-Ar), 127.45 (2x$\underline{C}$H-Ar), 78.06 ($\underline{C}$(CH$_3$)$_3$), 72.18 ($\underline{C}$H$_2$Ph), 69.31 (Cβ-Ser), 60.73 ($\underline{C}$H$_2$CH$_3$), 52.23 (Cα-Ser), 49.51 (Cα-Ala), 28.16 (C($\underline{C}$H$_3$)$_3$), 18.15 (Cβ-Ala), 13.98 (CH$_2\underline{C}$H$_3$). ESI-MS $[C_{20}H_{30}N_2O_6 + H]^+$ calcd 395.2, found 395.3

***N*-Boc-D-Ala-L-Ser-OEt (39).** *N*-Boc-D-alanyl-*O*-benzyl-L-serine ethyl ester (**38**) (2.34 g; 5.90 mmol) was stirred in presence of Pd/C (10 wt-% on carbon, 700 mg; 0.66 mmol) in MeOH under a hydrogen atmosphere (1 atm) at rt. After 14 h the reaction mixture was filtered over celite and volatiles were removed in vacuo. Purification by flash column chromatography (SiO_2; CH_2Cl_2 to CH_2Cl_2/MeOH = 10:1) yielded the ethyl ester **39** (1.54 g; 5.08 mmol; 86%) as colorless solid. ^{1}H NMR (500 MHz, DMSO-d_6) δ 7.92 (d, *J* = 7.9 Hz, 1H, N$\underline{H}$-Ser), 6.98 (d, *J* = 7.9 Hz, 1H, N$\underline{H}$-Ala), 5.06 (t, *J* = 5.5 Hz, 1H, O$\underline{H}$-Ser), 4.35 – 4.25 (m, 1H, Hα-Ser), 4.08 (q, *J* = 7.1 Hz, 2H, $C\underline{H}_2CH_3$), 4.03 (q, *J* = 7.4 Hz, 1H, Hα-Ala), 3.69 (dt, *J* = 10.8, 5.4 Hz, 1H, Hβ-Ser), 3.58 (dt, *J* = 10.9, 4.9 Hz, 1H, Hβ-Ser), 1.38 (s, 9H, $C(C\underline{H}_3)_3$), 1.21 – 1.11 (m, 6H, $CH_2C\underline{H}_3$, Hβ-Ala). ^{13}C NMR (126 MHz, DMSO-d_6) δ 172.91 (CO), 170.41 (CO), 155.02 (CO), 78.10 ($\underline{C}(CH_3)_3$), 61.33 (Cβ-Ser), 60.51 ($\underline{C}H_2CH_3$), 54.51 (Cα-Ser), 49.61 (Cα-Ala), 28.19 ($C(\underline{C}H_3)_3$), 18.21 (Cβ-Ala), 14.04 ($CH_2\underline{C}H_3$). ESI-MS $[C_{13}H_{24}N_2O_6 + H]^+$ calcd 305.2, found 305.2.

***N*-Boc-D-Ala-Dha-OEt (40).** *N*-Boc-D-alanyl-L-serine ethyl ester (**39**) (1.54 g; 5.06 mmol), and EDC*HCl (1.94 g; 10.1 mmol) was dissolved in anhydrous CH_2Cl_2 (100 mL). After freezing the solution, copper(I) chloride (50 mg; 0.50 mmol), was added and the mixture was degassed via freeze-thawing cycles (3 × 0.01 mbar 30 min/N_2 flush). The reaction mixture was then allowed to warm to rt and stirred under light exclusion and argon atmosphere for 22 h. After dilution with CH_2Cl_2 (100 mL) the organic layer was washed with satd. NH_4Cl solution (50 mL), dried over Na_2SO_4, filtered and the volatiles were removed in vacuo. Purification by flash column chromatography (SiO_2; PE to PE/EtOAc = 4:1) yielded the ethyl ester **40** (1.36 g; 4.74 mmol; 94%) as colorless oil. The title compound **40** was used immediately in the next step, due to observed high polymerization susceptibility. ^{1}H NMR (500 MHz, DMSO-d_6) δ 9.11 (s, 1H, N$\underline{H}$-DhA), 7.31 (d, *J* = 7.3 Hz, 1H, N$\underline{H}$-Ala), 6.24 (s, 1H, Hβ-DhA), 5.70 (s, 1H, Hβ-DhA), 4.22 (q, *J* = 7.1 Hz, 2H, $C\underline{H}_2CH_3$), 4.11 (p, *J* = 7.1 Hz, 1H, Hα-Ala), 1.38 (s, 9H, $C(C\underline{H}_3)_3$), 1.25 (t, *J* = 7.1 Hz, 3H, $CH_2C\underline{H}_3$), 1.20 (d, *J* = 7.2 Hz, 3H, Hβ-Ala). ^{13}C NMR (126 MHz, DMSO-d_6) δ 172.47 (CO), 163.28 (CO), 155.33 (CO), 132.34 (Cα-DhA), 108.36 (Cβ-DhA), 78.42 ($\underline{C}(CH_3)_3$), 61.64 ($\underline{C}H_2CH_3$), 50.33 (Cα-Ala), 28.14 ($C(\underline{C}H_3)_3$), 17.32 (Cβ-Ala), 13.92 ($CH_2\underline{C}H_3$). ESI-MS $[C_{13}H_{22}N_2O_5 + H]^+$ calcd 287.2, found 287.1.

***N*-Boc-D-Ala-Dha (41).** To a solution of *N*-Boc-D-Ala-Dha-OEt (**40**) (1.34 g; 4.68 mmol) in a THF/H_2O mixture (45 mL, 2:1), 1 M LiOH in H_2O (15 mL) was added and the reaction mixture was stirred for 30 min at rt. The colorless solution was cooled to 0 °C, acidified with aqueous HCl (1 M) to pH = 4 and extracted with CH_2Cl_2 (3 × 250 mL). The combined organic layers were dried over Na_2SO_4, filtered and the volatiles were removed in vacuo. Purification by flash column chromatography (SiO_2; CH_2Cl_2 to CH_2Cl_2/MeOH = 10:1) yielded the free acid **41** (1.15 g; 4.45 mmol; 95%) as a colorless foam. ^{1}H NMR (300 MHz, $CHCl_3$-d_1) δ 9.46 (bs, 1H, COO$\underline{H}$), 8.73 (s, 1H, N$\underline{H}$-DhA), 6.65 (s, 1H, N$\underline{H}$-Ala), 6.00 (s, 1H, Hβ-DhA), 5.52 (d, *J* = 8.7 Hz, 1H, Hβ-DhA), 4.91 – 4.55 (m, 1H, Hα-Ala), 1.63 – 1.26 (m, 12H, Hβ-Ala, $C(C\underline{H}_3)_3$). ^{13}C NMR (75 MHz, $CHCl_3$-d_1) δ 172.50 (CO), 166.39 (CO), 156.12 (CO), 131.09 (Cα-DhA), 110.61 (Cβ-DhA), 81.19 ($\underline{C}(CH_3)_3$), 50.54 (Cα-Ala), 28.43 ($C(\underline{C}H_3)_3$), 19.25 (Cβ-Ala). ESI-MS $[C_{11}H_{18}N_2O_5 + Na]^+$ calcd 281.1, found 281.1.

***N*-Boc-Sar-SNAc (43).** Boc-sarcosine (**42,** 1.32 g; 6.99 mmol) was dissolved in anhydrous CH_2Cl_2 (70 mL) and cooled to 0 °C under nitrogen atmosphere. DIPEA (2.37 mL; 14.0 mmol) and subsequently Fluoro-*N,N,N',N'*-tetramethylformamidinium hexafluorophosphate (2.03 g; 7.70 mmol) were added and the reaction mixture was stirred for 1 h at 0 °C. *N*-Acetylcysteamine (0.89 mL; 8.39 mmol) was then added drop-wise and the reaction mixture was stirred for an additional 1 h at 0 °C. The reaction mixture was washed with satd. $NaHCO_3$ solution and the aqueous phase was extracted with CH_2Cl_2 (5 × 30 mL). The combined organic layers were dried over Na_2SO_4, filtered and the volatiles were removed in vacuo. Purification by flash column chromatography (SiO_2; CH_2Cl_2 to CH_2Cl_2/MeOH = 10:1) yielded the impure title compound which was further purified by preparative HPLC (Nucleodur C18 Gravity-SB, VP250 * 21 mm, 5 µM (Machery Nagel); Eluent A: H_2O; Eluent B: MeCN; isocratic gradient from 25% B to 40% B during 30 min; flow 10 mL/min). After lyophilisation of the product containing fractions, the colorless oil was coevaporated with toluene (3 × 100 mL) to remove residual water. The Boc protected SNAc-ester **43** (1.56 g; 5.32 mmol; 76%) was isolated as a colorless oil. ^{1}H NMR (500 MHz, DMSO-d_6) two rotamers: δ 8.05 (t, J = 5.7 Hz, 1H, $N\underline{H}Ac$), 4.11 (s, 1H, Hα-$Sar_{rotamer1}$), 4.09 (s, 1H, Hα-$Sar_{rotamer2}$), 3.21 – 3.11 (m, 2H, $SCH_2C\underline{H}_2NHAc$), 2.96 – 2.89 (m, 2H, $SC\underline{H}_2CH_2NHAc$), 2.88 (s, 3H, $NC\underline{H}_3$-$Sar_{rotamer1}$), 2.86 (s, 3H, $NC\underline{H}_3$-$Sar_{rotamer2}$), 1.78 (s, 3H, $C\underline{H}_3$-NHAc), 1.41 (s, 9H, $C(C\underline{H}_3)_{3\ rotamer1}$), 1.34 (s, 9H, $C(C\underline{H}_3)_{3\ rotamer2}$). ^{13}C NMR (126 MHz, DMSO-d_6) two rotamers: δ 198.12 ($\underline{C}OS_{rotamer2}$), 197.97 ($\underline{C}OS_{rotamer1}$), 169.24 ($\underline{C}O$-$NHAc_{rotamer1}$), 169.20 ($\underline{C}O$-$NHAc_{rotamer2}$), 155.08 ($O\underline{C}OC(CH_3)_{3\ rotamer1}$), 154.36 ($O\underline{C}OC(CH_3)_{3\ rotamer2}$), 79.59 ($\underline{C}(CH_3)_{3\ rotamer1}$), 79.46 ($\underline{C}(CH_3)_{3\ rotamer2}$), 58.54 (Cα-$Sar_{rotamer2}$), 57.96 (Cα-$Sar_{rotamer1}$), 38.19 ($SCH_2\underline{C}H_2NHAc_{rotamer2}$), 38.10 ($SCH_2\underline{C}H_2NHAc_{rotamer1}$), 35.72 ($N\underline{C}H_3$-$Sar_{rotamer1}$), 35.54 ($N\underline{C}H_3$-$Sar_{rotamer2}$), 27.98 ($C(\underline{C}H_3)_{3\ rotamer1}$), 27.81 ($C(\underline{C}H_3)_{3\ rotamer2}$), 27.44 ($S\underline{C}H_2CH_2NHAc_{rotamer1}$), 27.39 ($S\underline{C}H_2CH_2NHAc_{rotamer2}$), 22.51 ($\underline{C}H_3$-NHAc). ESI-MS $[C_{12}H_{22}N_2O_4S + H]^+$ calcd 291.1, found 291.1.

Sar-SNAc * HCl (44). *N*-Boc-sarcosine SNAc ester (**43**) was dissolved in anhydrous 1,4-dioxane (10 mL), 7 mL 4 M HCl in dioxane was added and the reaction mixture was stirred for 4 h at rt. The colorless suspension was filtered and the precipitant was washed with cold Et_2O (3 × 5 mL). The hydrochloride **44** (1.15 g; 5.07 mmol; 99%) was isolated as a colorless solid. ^{1}H NMR (500 MHz, DMSO-d_6) δ 9.61 (q, J = 5.9 Hz, 2H; $N\underline{H}_2$-Sar), 8.30 (t, J = 5.7 Hz, 1H, $N\underline{H}Ac$), 4.21 (t, J = 5.8 Hz, 2H, Hα-Sar), 3.20 (q, J = 6.4 Hz, 2H, $SCH_2C\underline{H}_2NHAc$), 3.03 (t, J = 6.7 Hz, 2H, $SC\underline{H}_2CH_2NHAc$), 2.51 (t, J = 5.3 Hz, 3H, $NC\underline{H}_3$-Sar), 1.79 (s, 3H, $C\underline{H}_3$-NHAc). ^{13}C NMR (126 MHz, DMSO-d_6) δ 192.62 ($\underline{C}OS$), 169.55 ($\underline{C}O$-NHAc), 55.20 (Cα-Sar), 37.93 ($SCH_2\underline{C}H_2NHAc$), 32.47 ($N\underline{C}H_3$-Sar), 28.20 ($S\underline{C}H_2CH_2NHAc$), 22.57 ($\underline{C}H_3$-NHAc). ESI-MS $[C_7H_{14}N_2O_2S + H]^+$ calcd 191.1, found 191.0.

***N*-Boc-D-Ala-Dha-Sar-SNAc (35b).** To a solution of *N*-Boc-D-Ala-Dha-OH (**41**) (155 mg; 0.60 mmol) and DIPEA (306 µL; 1.80 mmol) in anhydrous CH_2Cl_2 (20 mL), bis(2-oxo-3-oxazolidinyl)phosphinic chloride (153 mg; 0.60 mmol) was added at 0 °C and the reaction mixture was stirred for 30 min under a nitrogen atmosphere. The SNAc-ester **44** (136 mg; 0.60 mmol) was added and the reaction mixture was stirred to rt over night. EtOAc (60 mL) was added and the organic phase was washed with satd. $NaHSO_3$ (2 × 10 mL), satd. $NaHCO_3$

(2 × 10 mL) and brine (2 × 10 mL). The organic layer was dried over Na_2SO_4, filtered and the volatiles were removed in vacuo. Purification by flash column chromatography (SiO_2; CH_2Cl_2 to CH_2Cl_2/MeOH = 10:1) yielded the Boc-protected SNAc-ester **35b** (174 mg; 0.40 mmol; 67%) as a colorless foam. ^{1}H NMR (300 MHz, MeOH-*d₄*) δ 5.49 (s, 3H, NMe), 5.34 (s, 1H, 1H of Dha-CH2, major), 5.20 (s, 1H, 1H of Dha-CH2, minor), 4.91 (s, 1H, 1H of Dha-CH2, major), 4.62 – 4.16 (m, 2H, Sar-CH2), 4.10 (q, J = 7.2 Hz, 1H, Ala-Hα), 3.35 (t, J = 6.6 Hz, 2H, SNAc-CH2, major), 3.15 (s, 2H, SNAc-CH2, minor), 3.07 (t, J = 6.5 Hz, 2H, SNAc-CH2, major), 3.01 (br s, 2H, SNAc-CH2, minor), 2.01 (s, 3H, Ac, minor), 1.92 (s, 3H, Ac, major), 1.44 (s, 9H, Boc), 1.32 (d, J = 7.2 Hz, 3H, Ala-Hβ). ^{13}C NMR (126 MHz, MeOH-d_4) δ 197.59 (RCO-SR), 173.90, 173.42, 170.06 (3C, RCO-N), 157.60 (O-CO-O,) 138.12 (Dha-Cα), 103.83 (Dha-Cβ), 80.61 ((CH3)3C-), 61.51 (Sar-CH2, major), 58.08 (Sar-CH2, minor), 54.80 (NMe), 51.49 (Ala-Cα), 39.88 (SNAc-CH2, major), 39.19 (SNAc-CH2, minor), 28.85 (SNAc-CH2, major), 28.69 (Boc-CH3), 22.54 (Ac, major), 20.88 (Ac, minor), 18.09 (Ala-Hβ). ESI-MS $[C_{18}H_{30}N_4O_6S+H]^+$ calcd 431.2, found 431.2.

D-Ala-Dha-Sar-SNAc (14). *N*-Boc-D-Alanyl-dehydroalanyl-sarcosine SNAc ester (**35b**) (100 mg; 0.23 mmol) was suspended in 1,4-dioxane (9 mL) and 4 M HCl in 1,4-dioxane (9 mL) was added. The reaction mixture was stirred for 18 h at r.t. and the suspension was filtered. The colorless solid was washed with 1,4-dioxane (4 × 2 mL), cold Et_2O (4 × 2 mL) and dried in vacuo to yield the hydrochloride salt of **14** (83 mg; 2.3 mmol; 99%) as a colorless solid. HPLC-MS: column EC150/2 Nucleoshell HILIC 2.7 μM (Macherey-Nagel, Germany), buffer A: 100 mM ammonium formate buffer B: MeCN / 0.1% HCO_2H, gradient of 5%A to 12% A over 12 min (flow 800 μL/min). ESI-MS $[C_{13}H_{22}N_4O_4S + H]^+$ calcd 331.1, found 331.1. HR-MS $[C_{13}H_{22}N_4O_4S + H]^+$ calcd 331.1435, found 331.1438.

General methods for DNA manipulation, analysis and PCR

Plasmid DNA was either purified by standard alkaline lysis [4] or by using the GeneJet Plasmid Miniprep Kit (Thermo Fisher Scientific) or the NucleoBond PC100 kit (Machery Nagel). Oligonucleotides were obtained from Sigma-Aldrich. PCR reactions were carried out in a Mastercycler® pro (Eppendorf) using Phusion™ High-Fidelity according to the manufacturer's protocol. Initial denaturation (1 min, 98 °C); 30 cycles of denaturation (20 s, 98 °C), annealing (25 s, 53–64 °C) and elongation (varied based on PCR product length 0.5 kb/ min, 72 °C); and final extension (5 min, 72 °C). Restriction enzymes were purchased from Thermo Fisher Scientific. DNA fragments were separated by agarose gel electrophoresis and purified by gel extraction by NucleoSpin® Gel and PCR Clean-up (Macherey-Nagel) or ethanol precipitation (for the fragments larger than 10 kb). T4 DNA ligase (Thermo Fisher Scientific) was used for ligation. Shrimp alkaline phosphatase (Thermo Fisher Scientific) was used to treat the accept vectors for avoiding self-ligation. Plasmid preparations, restriction digestions, gel electrophoresis, and ligation reactions were carried out according to standard methods [4].

Construction of the block mutant *M. xanthus* DK1622 Δ*mchA-tet*::pArg345-V1

The *M. xanthus* DK1622 Δ*mchA-tet*::pArg345-V1 mutant, designed for mutasynthesis studies (Fig. S1), was constructed by deletion of the *arg2* gene from pArg2345-V1 (Pogorevc et al., unpublished data) expression construct. The expression construct was hydrolyzed using *Nde*I/*Sph*I restriction enzymes, to excise 10.1 kb *arg2* fragment, resulting in pArg345-V1 fragment. A 1097 bp amp^R cassette was amplified from pUC18 plasmid using the oligonucleotides Arg44 (TGACATCATATGCCTAGGGACGAAAGGGCC-TCGTGATAC) and Arg45 (ATGTCAGCATGCCCTAGGTTACCAATGCTTAATCAGTGAG). The PCR fragment was hydrolyzed using *Nde*I/*Sph*I restriction enzymes and ligated into previously generated pArg345-V1 fragment, producing pArg345-V1-amp plasmid, which was hydrolyzed using *Aar*I restriction enzyme and subsequently re-ligated to construct pArg345-V1. The heterologous host *M. xanthus* DK1622 Δ*mchA-tet* was transformed with the modified expression construct by electroporation according to established procedures [5]. Correct mutants were selected on CTT agar supplemented with 50 µg/mL kanamycin and genotypic verification of the mutants was performed according to the previously described procedure (Pogorevc et al., unpublished data). The obtained mutant was cultivated in comparison to the control strain *M. xanthus* DK1622 *ΔmchA-tet::*pArg2345-V1 and argyrin production was evaluated by HPLC-MS analysis of the culture extracts (see supporting information). Analysis revealed abolishment of the argyrin production in the *M. xanthus* DK1622 Δ*mchA-tet*::pArg345-V1 mutant.

Feeding of mutasynthons to *M. xanthus* DK1622 Δ*mchA-tet*::pArg345-V1

Mimics of the native tripeptide intermediates (SNAc-tripeptides) were fed to cultures of the mutant *M. xanthus* DK1622::pArg345-V1. Mutant strains of *Myxococcus xanthus* DK1622 were routinely cultivated in CTT medium (10 g/L casitone; 10 mM Tris-HCl, pH 8.0; 8 mM $MgSO_4$; 10 mM potassium phosphate, pH 7.6) or M7/s4 medium (0.5% soy flour, 0.5% corn starch, 0.2% glucose, 0.1% yeast extract, 0.1% $MgSO_4 \times 7\ H_2O$, 0.1% $CaCl_2 \times 2\ H_2O$, 1% HEPES, with final pH 7.4 and supplemented with 0.1 mg/L of vitamin B12 and 5 mg/L of $FeCl_3$ after autoclaving) amended with 50 µg/mL kanamycin for *M. xanthus* DK1622 Δ*mchA-tet::*pArg345-V1 and 50 µg/mL kanamycin plus 50 µg/mL zeocin for *M. xanthus* DK1622 Δ*mchA-tet::*pArg345-V1/pDKzeo1-nptII-arg2. Cultivations were carried out in shake flasks on a rotary shaker at 200 rpm and at 30 °C. Feeding experiments were performed in 20 mL scale. 15 mL production medium was inoculated with 5 mL of a well grown pre-culture of *M. xanthus* DK1622::pArg345-V1. Mutasynthons were fed in equal portions (50 µL each) after 24 h, 27 h, 30 h, 33 h and 36 h from a DMSO stock solution of the synthesized tripeptide thioesters (12 mg/mL). After 39 h the adsorber resin XAD-16 was added (2% final conc.) and cultivation was continued overnight. The cultures were harvested by centrifugation at 8.000 rpm for 10 min and cells/XAD were extracted with 40 mL MeOH (stirring for 60 min at room temperature followed by filtration). Evaporated extracts were re-dissolved in 200 µL MeOH for HPLC-MS analysis. Feeding studies included parallel processing of a culture to which a pure DMSO solution (without mutasynthon) was supplied. Argyrin could be detected in pellet of *M. xanthus* DK1622 Δ*mchA-tet::*pArg345-V1/pDKzeo1-nptII-arg2, but not in pellet of *M. xanthus* DK1622 Δ*mchA-tet::*pArg345-V1 fed with synthetic tripeptide.

In order to evaluate stability of the synthetic tripeptide mimic, its degradation kinetics was evaluated under the standard cultivation conditions. Two mL Eppendorf tube was filled with two mL of the cultivation medium supplemented with 25 µL of the tripeptide DMSO stock solution (12 mg/mL) and incubated at 30 °C, 800 rpm in the heat block. A 100 µL sample was taken every 2 h, centrifuged at 15000 rpm, 4 °C for 5 min and supernatant was subjected to HPLC-MS analysis: column EC150/2 Nucleoshell HILIC 2.7 µM (Macherey-Nagel, Germany), buffer A: 100 mM ammonium formate buffer B: MeCN / 0.1% HCO_2H, gradient of 5%A to 12% A over 12 min (flow 800 µL/min), (Fig. S2).

Co-expression of *arg2* in *M. xanthus* DK1622 Δ*mchA-tet*::pArg345-V1

Plasmid pGH-arg2-V1 (Pogorevc et al., unpublished data) was hydrolyzed by *Hind*III/*Xba*I to excise *arg2* fragment which was subsequently ligated into pDKzeo1[6] plasmid hydrolyzed with the same enzymes, to produce pDKzeo1-arg2-V1. To introduce P_{nptII} promoter upstream of the *arg2* gene, a 208 bp fragment was amplified from pArg2345-V1 using oligonucleotides Arg47 (CACCAAGCTTTGAA-TGCGCAAACCAACC) and Arg48 (CTAGGTCAGGGCATATGATC). The resulting P_{nptII} promoter fragment and the expression plasmid pDKzeo1-arg2-V1 were hydrolyzed by *Hind*III/*Nde*I restriction enzymes and ligated to produce pDKzeo1-nptII-arg2-V1. The heterologous host *M. xanthus* DK1622 Δ*mchA-tet::*pArg345-V1 was transformed with the generated expression construct by electroporation. Correct mutants were selected on CTT agar supplemented with 50 µg/mL zeocin plus 50 µg/mL kanamycin and genotypic verification of the mutants was performed according to the previously described procedure (Pogorevc et al., unpublished data). The obtained mutant was cultivated in comparison to the control strain *M. xanthus* DK1622 *ΔmchA-tet::*pArg2345-V1 and argyrin production was evaluated by HPLC-MS analysis of the culture extracts. The mutant strain *M. xanthus* DK1622 Δ*mchA-tet::*pArg345-V1/pDKzeo1-nptII-arg2 showed the same argyrin production profile as the control strain.

In vitro reconstitution of argyrin using cell lysate

The mutant expressing Arg2 subunit (DK1622 Δ*mchA-tet::*pDKzeo1-nptII-arg2) was generated by transformation of previously generated construct pDKzeo1-nptII-arg2 into the DK1622 Δ*mchA-tet* heterologous host. Correct mutants were selected on CTT agar supplemented with 50 µg/mL zeocin and genotypic verification of the mutants was performed according to the previously described procedure (Pogorevc *et al.*, unpublished data). The mutant strains DK1622 Δ*mchA-tet::*pDKzeo1-nptII-arg2 and *M. xanthus* DK1622 Δ*mchA-tet::*pArg345-V1 were cultivated in CTT medium supplemented with 50 µg/mL zeocin and 50 µg/mL kanamycin, respectively. After 24–48 h of cultivation or when the culture OD_{600} reached around 1.0, the cultures were harvested by centrifugation at 10000g, 4 °C for 15 min. Cell pellets were washed with brine twice, before cells were resuspended in lysis buffer (2 mM DTT, 50 mM Tris-HCl (pH 7.5), 150 mM NaCl, 10 mM $MgCl_2$) and disrupted by sonication. Two mL of lysis buffer was used per 1 g of cell pellet. The homogenate was clarified by centrifugation at 80000g, 4 °C, 10 min. Total protein concentration in cell lysates was measured by nano drop. In vitro assay was performed in total volume of 50 µL containing reaction

buffer (50 mM Tris-HCl (pH 7.5 or 8.0), 150 mM NaCl, 10 mM $MgCl_2$), 3 mM ATP, 1 mM substrate amino acid mixture (alanine, serine, glycine, cysteine, tryptophan) and a mixture of both cell lysates containing Arg2 and Arg3-Arg5 proteins, respectively. Varying volumes of cell lysate mixture were used, ranging from 0.05 mg to 0.8 mg of total protein content. In vitro reconstitution was performed by incubation for 2 h or 4 h at room temperature (22 °C) or 30 °C. After incubation the samples were extracted by addition of 50 µL MeOH, centrifuged at 4 °C for 15 min and the supernatant was analysed by HPLC–MS.

HPLC–MS method for analysis of argyrin mutasynthesis and *in vitro* reconstitution samples

All measurements were performed on a Dionex Ultimate 3000 RSLC system using a Waters BEH C18, 50 × 2.1 mm, 1.7 µm dp column. Separation of 1 µL sample was achieved by a linear gradient with (A) H_2O + 0.1% FA to (B) ACN + 0.1% FA at a flow rate of 600 µL/min and 45 °C. The gradient was initiated by a 1 min isocratic step at 5% B, followed by an increase to 95% B in 6 min to end up with a 1.5 min step at 95% B before reequilibration under the initial conditions. UV spectra were recorded by a DAD in the range from 200 to 600 nm. The LC flow was split to 75 µL/min before entering the maXis 4G hr-ToF mass spectrometer (Bruker Daltonics, Bremen, Germany) using the standard ESI source. Mass spectra were acquired in centroid mode ranging from 150 – 2000 *m*/*z* at a 2 Hz scan speed.

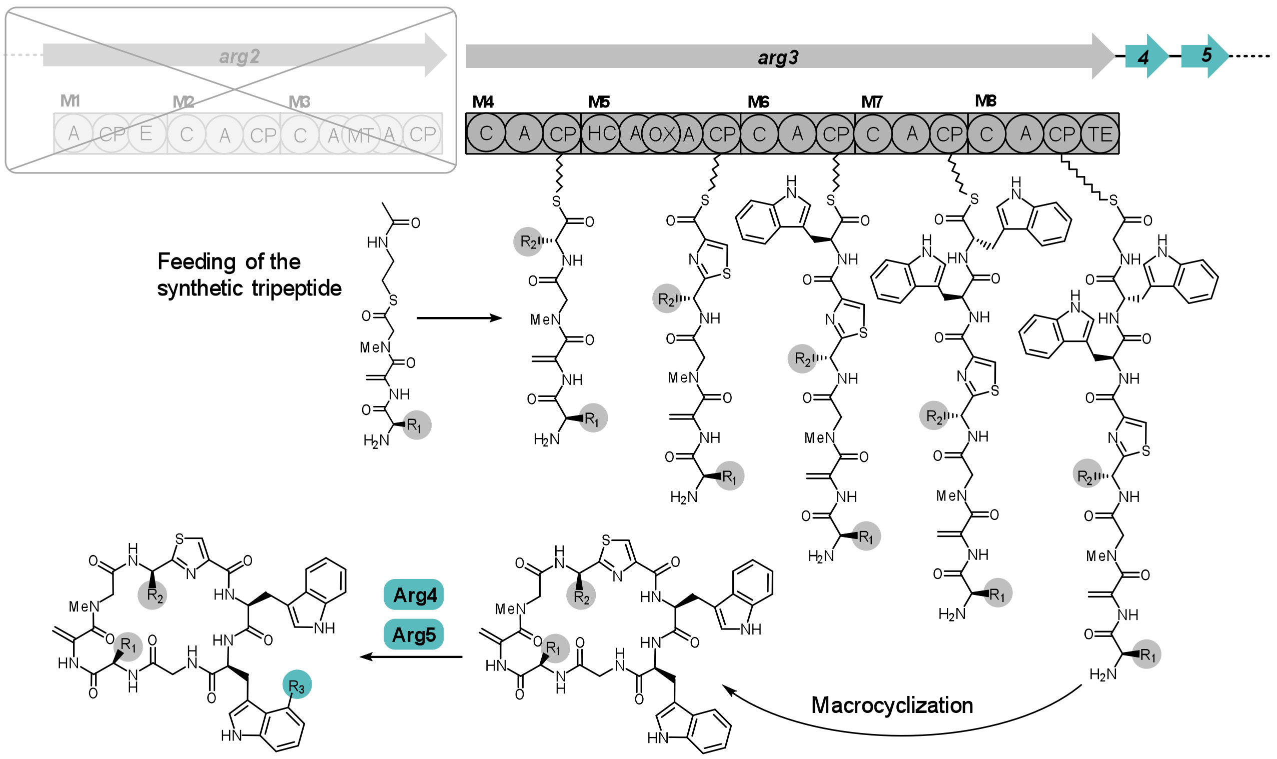

Figure S1. Mutasynthesis approach for production of argyrins. An expression strain harboring a truncated *arg* BGC lacking *arg1* and *arg2* (encoding the first subunit Arg2 of the argyrin NRPS megasynthetase) is fed with mutasynthons to restore argyrin production. Mutasynthons represent mimics of natural or unnatural tripeptide intermediates.

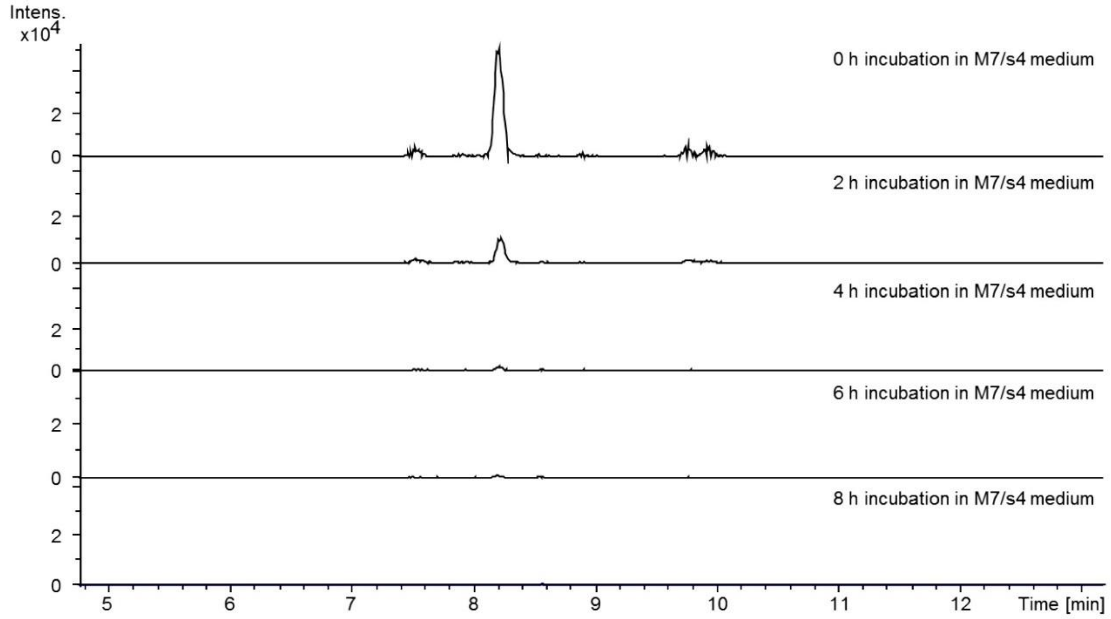

Figure S2. Degradation kinetics of the D-Ala-Dha-Sar-SNAc mutasynthon **14** in M7/S4 medium over 8 h period. The chromatogram shows EIC $[M + H]^+ = 331.143$.

References

1. Robins, M. J., Peng, Y., Damaraju, V. L., Mowles, D., Barron, G., Tackaberry, T., Young, J. D., and Cass, C. E. Improved syntheses of 5'-S-(2-aminoethyl)-6-N-(4-nitrobenzyl)-5'- thioadenosine (SAENTA), analogues, and fluorescent probe conjugates: analysis of cell-surface human equilibrative nucleoside transporter 1 (hENT1) levels for prediction of the antitumor efficacy of gemcitabine. *J. Med. Chem.* **53(16)**, 6040-53 (2010).

2. Dahiya, R. Synthesis and in vitro cytotoxic activity of a natural peptide of plant origin. *J. Iran. Chem. Soc.* **5(3)**, 445–452 (2008).

3. Kanemitsu, T., Ogihara, Y., and Takeda, T. Synthetic Studies on Glycopeptides Concerned with Defense Response of Plants. I. Syntheses of Supprescins A and B. *Chem. Pharm. Bull.* **45(4)**, 643-650 (1997).

4. Sambrook, J.F. and Russel, D. W. (eds.) *Molecular Cloning: A Laboratory Manual.* Cold Spring Harbor Laboratory Press; 2001.

5. Kashefi, K. and Hartzell, P. L. Genetic suppression and phenotypic masking of a Myxococcus xanthus frzF- defect. *Mol. Microbiol.* **15(3)**, 483-94 (1995).

6. Krug, D. and Müller, R. Discovery of additional members of the tyrosine aminomutase enzyme family and the mutational analysis of CmdF. *ChemBioChem* **10(4)**, 741-50 (2009).
